# Exploring Pharmacological Mechanisms of Xuefu Zhuyu Decoction in the Treatment of Traumatic Brain Injury via a Network Pharmacology Approach

**DOI:** 10.1155/2018/8916938

**Published:** 2018-10-04

**Authors:** Yuanyuan Zhong, Jiekun Luo, Tao Tang, Pengfei Li, Tao Liu, Hanjin Cui, Yang Wang, Zebing Huang

**Affiliations:** ^1^Institute of Integrative Medicine, Xiangya Hospital, Central South University, Changsha 410008, China; ^2^Department of Gerontology, Traditional Chinese Medicine Hospital Affiliated to Xinjiang Medical University, Urumqi 830000, China; ^3^Department of Infectious Disease, Hunan Key Laboratory of Viral Hepatitis, Xiangya Hospital, Central South University, Changsha 410008, China

## Abstract

**Objectives:**

Xuefu Zhuyu decoction (XFZYD), a traditional Chinese medicine (TCM) formula, has been demonstrated to be effective for the treatment of traumatic brain injury (TBI). However, the underlying pharmacological mechanisms remain unclear. This study aims to explore the potential action mechanisms of XFZYD in the treatment of TBI and to elucidate the combination principle of this herbal formula.

**Methods:**

A network pharmacology approach including ADME (absorption, distribution, metabolism, and excretion) evaluation, target prediction, known therapeutic targets collection, network construction, and molecule docking was used in this study.

**Results:**

A total of 119 bioactive ingredients from XFZYD were predicted to act on 47 TBI associated specific proteins which intervened in several crucial pathological processes including apoptosis, inflammation, antioxidant, and axon genesis. Almost each of the bioactive ingredients targeted more than one protein. The molecular docking simulation showed that 91 pairs of chemical components and candidate targets had strong binding efficiencies. The “Jun”, “Chen”, and “Zuo-Shi” herbs from XFZYD triggered their specific targets regulation, respectively.

**Conclusion:**

Our work successfully illuminates the “multicompounds, multitargets” therapeutic action of XFZYD in the treatment of TBI by network pharmacology with molecule docking method. The present work may provide valuable evidence for further clinical application of XFZYD as therapeutic strategy for TBI treatment.

## 1. Introduction

Traumatic brain injury (TBI) is a major cause of death and disability [1]. At least 10 million severe TBIs result in death or hospitalization annually worldwide [2]. Approximately 1.7 million Americans sustain a TBI each year, leading to over 1.4 million emergency department visits, 275 000 hospital admissions, and 50 000 deaths that contribute to one-third of all injury-related deaths [3]. In the European Union alone, an estimated 1.5 million hospital admissions and 57,000 deaths annually attribute to TBI [4]. In China, TBI-related mortality remains a high level, ranging from 12.99 to 17.06 per population of 100 000 persons [5]. Thus, TBI has afforded huge social and economic burden.

TBI is a diverse group of sterile injuries induced by primary and secondary mechanisms that give rise to cell death, inflammation, and neurologic dysfunction in patients of all demographics [6, 7]. The primary injury is caused by the mechanical stress or shear force on tissues with no therapeutic agents [8]. The secondary injury includes a wide variety of processes like activation of inflammatory and immune response [9, 10], calcium overload [11], glutamate toxicity [12], and mitochondrial dysfunction [13], among others. Current guidelines for the management of the secondary injury are primarily supportive, including the emphasis on surveillance (i.e., intracranial pressure) and the preventive measures to reduce morbidity and mortality [14]. Despite the fact that detailed medicines contain free-radical scavengers, antagonists of N-methyl-D-aspartate, and calcium channel blockers [15], the results of the controlled clinical trials of these drugs are disappointing [16]. Neuroscientists and doctors tend to search for potential novel drugs from traditional Chinese medicine (TCM) library to treat TBI [17].

TCM is a comprehensive medicinal system that has been used in clinical practice for thousands of years and plays an important role in the health maintenance for people all over the world [18, 19]. The validated curative effects of TCM make it a feasible alternative therapeutic agent for disease treatment. Xuefu Zhuyu decoction (XFZYD), a representative TCM formula, was first recorded in* Correction of Errors in Medical Works* by Qing-ren Wang. XFZYD consists of 11 crude herbs:* Persicae Semen (Tao ren), Carthami Flos (Hong hua) Radix Paeoniae Rubra (Chi shao), Chuanxiong Rhizoma (Chuan xiong), Achyranthis Bidentatae Radix (Niu xi), Angelicae Sinensis Radix (Dang gui), Rehmannia glutinosa Libosch (Sheng di huang), Platycodon Grandiforus (Jie geng), Aurantii Fructus (zhi qiao), Radix Bupleuri (chai hu), and licorice (Gan cao).* The main chemicals from XFZYD include flavonoids, organic acids, terpenoids, and steroidal saponins [20–22]. The formula has been proven reliable and effective for curing various diseases including unstable angina pectoris [23, 24], coronary artery disease [25], thromboembolic stroke [26], ischemic stroke [27], and TBI. The therapeutic agent of XFZYD is to promote blood circulation and remove blood stasis according to the TCM theory. Several randomized controlled clinical trials and animal experiments have showed definite therapeutic effects of XFZYD for the treatment of TBI [28–31]. Recent researches demonstrate that XFZYD provides neuroprotection via anti-inflammatory pathway and cognitive improvement through synaptic regulation [32, 33]. However, merely these evidences to explain the multiple therapeutic mechanisms of TCM for TBI treatment are unavailable. Because the effects of TCM are always controversial in terms of their abstract theory, unclear basis, complex interactions between various ingredients, and complex interactive biological systems [25], it is essential to develop an advanced technique to deeply uncover the synthesized pharmacological effects of XFZYD in the treatment of TBI.

With the development of TCM modernization, network pharmacology has become a novel method to elucidate the multi-druggable targets effects of TCM [34]. TCM network pharmacology, first proposed by Shao Li [35], makes it feasible to understand the effective constituents and targets of the herbs from TCM formula. This analytical method integrates bioinformatics, systems biology, and polypharmacology and further utilizes network analysis to imply the multiple actions of drugs across multiple scales ranging from molecular/cellular to tissue/organism levels [36, 37]. Coinciding with the holistic and systemic characteristics of TCM, network pharmacology is expected to bridge the gap between TCM and modern medicine [25]. Previous researches have clarified the scientific basis and systematic features of herbal medicine to treat diseases through network pharmacology such as Qing-Luo-Yin and Ma-Huang Decoction etc. [38, 39].

**Figure 1:**
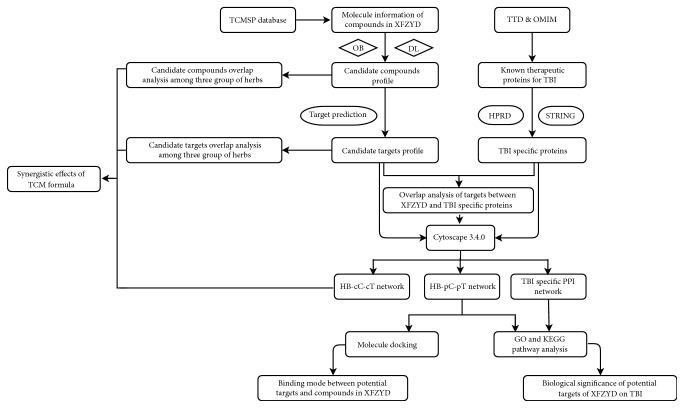
A schematic diagram of the network pharmacology-based strategies for determining the pharmacological mechanisms of the herbal formula XFZYD on TBI.

In the present work, we explored the pharmacological mechanisms of XFZYD acting on TBI via a network pharmacology approach. Network analyses and molecular docking method were used to reveal candidate drug targets related to TBI. Target analysis suggested that XFZYD regulated several key biological processes of TBI development such as apoptosis, inflammation, blood coagulation, and axon genesis. These processes contributed to the clarifying of the molecular mechanisms of XFZYD for TBI treatment. This will help to improve the effectiveness and specificity of TCM clinical usage (Figure 1 depicts a flowchart of the entire research procedure).

## 2. Methods

### 2.1. Database Construction

The chemical ingredients of 11 herbs in XFZYD were screened from Traditional Chinese Medicine Systems Pharmacology database (TCMSP, http://lsp.nwu.edu.cn/tcmsp.php) [40]. As a chemically oriented herbal encyclopedia, TCMSP can provide comprehensive information about herb ingredients including chemical structural data, oral bioavailability, drug targets, and their relationships with diseases, as well as the biological or physiological consequences of drug actions involving drug-likeness, intestinal epithelial permeability, and aqueous solubility [40]. The structures of these compounds were saved as mol2 format for further analysis. Discovery studio 2.5 was employed to optimize these molecules with a Merck molecular force field (MMFF). All detailed information about these ingredients is provided in Table S1.

### 2.2. Pharmacokinetic Prediction

Due to the disadvantages of biological experiments as being time-consuming and of high cost, identification of ADME (absorption, distribution, metabolism, and excretion) properties by in silico tools has now become a necessary paradigm in pharmaceutical research. In this study, 2 ADME-related models, including the evaluation of oral bioavailability (OB) and drug-likeness (DL), were employed to identify the potential bioactive compounds of XFZYD.

Oral bioavailability (OB), one of the most important pharmacokinetic parameters, represents the speed of a drug of becoming available to the body and the eventually absorbed extent of the oral dose [41], which is particularly significant in drug discovery of TCM for most oral Chinese herb formulas. Poor OB is indeed the main reason responsible for the unsuccessful development of compounds into therapeutic drugs in drug screening cascades. Here, a reliable in silico model OBioavail 1.1 [42] which integrates the metabolism (P450 3A4) and transport (P-glycoprotein) information was employed to calculate the OB values of herbal ingredients. In this study, OB≥30% (a suggested criterion by TCMSP database) was regarded as one threshold for screening possible candidate drugs presently, while 2 compounds with OB ≤ 30% were also taken into consideration due to their therapeutic effects according to literatures, such as amygdalin and hydroxysafflor yellow A [43, 44].

Drug-likeness (DL) is a qualitative profile used in drug design to evaluate whether a compound is chemically suitable for drug, and how drug-like a molecule is with respect to parameters affecting its pharmacodynamic and pharmacokinetic profiles which ultimately impacts its ADME properties [45]. In this study, the drug-likeness (DL) index (see (1)) using the Tanimoto coefficient [46] was computed for each compound in XFZYD:

(1) $$\begin{array}{c} T\left(\;X,Y\;\right)=\frac{X\cdot Y}{{\left\|X\right\|}^{2}+{\left\|Y\right\|}^{2}-X\cdot Y} \end{array}$$

where X represents the molecular descriptors of herb compounds and Y is the average molecular properties of all compounds in Drugbank database (http://www.drugbank.ca/). Compounds with DL ≥0.18 (average value for Drugbank) were selected as bioactive compounds in XFZYD.

In summary, compounds with OB ≥30% and DL≥0.18 were selected for subsequent research and others were excluded. The criteria used here were mainly for (1) extracting information from the herbs as much as possible with the least number of components and (2) explaining the obtained model by the reported pharmacological data.

### 2.3. Target Prediction

To obtain the molecular targets of these active ingredients, an in-house developed model SysDT based on Random Forest (RF) and Support Vector Machine (SVM) methods [47] was proposed, which efficiently integrated large-scale information on chemistry, genomics, and pharmacology. This approach shows impressive performance of prediction for drug-target interactions, with a concordance of 82.83%, a sensitivity of 81.33%, and a specificity of 93.62%, respectively [40]. UniProtKB (http://www.uniprot.org/) was employed to obtain the standard name of the predicted target proteins.

### 2.4. TBI-Specific Protein Collecting

The known therapeutic target proteins of TBI were screened from Therapeutic Target Database (TTD, available http://bidd.nus.edu.sg/group/cjttd/). TTD is a publicly accessible database which provides comprehensive information about the known therapeutic protein and nucleic acid targets described in the literature, the targeted disease conditions, the pathway information, and the corresponding drugs/ligands directed at each of these targets [48]. We also searched for Online Mendelian Inheritance in Man database (OMIM, available: http://omim.org/) to get proteins related to TBI. OMIM catalogues all known diseases with a genetic component and then possibly links them to the relevant genes in the human genome and provides references for further research and tools for genomic analysis of a catalogued gene [49]. Then, the proteins acquired from both databases were used as hub proteins and submitted to Human protein Reference Database [50] (HPRD, available http://www.hprd.org/) and STRING [51] (https://string-db.org/) to generate the proteins interacting with these hub proteins. HPRD is a database containing curated proteomic information pertaining to human proteins. The human protein-protein interaction (PPI) data on HPRD (Release 9) consists of 39,240 interactions among 9617 genes. The STRING database provides both experimental and predicted interaction information, providing a probabilistic association confidence score.

### 2.5. Molecule Docking

The LibDock algorithm based on the CHARMm Force Field in Discovery Studio (DS) 2.5 was used in this study to evaluate the potential molecular binding mode between bioactive compounds and putative targets. The crystal structure of the target proteins of XFZYD for treating TBI was downloaded from the RCSB Protein Data Bank (www.rcsb.org). The 3D chemical structures of bioactive compounds were downloaded from PubMed Compound database or TCMSP database and subjected to minimize the energy by using molecular mechanics-2 (MM2) force field. The protein preparation protocol was used before docking such as inserting missing atoms in incomplete residues, removing water, and protonating titratable residues. The ligand preparation protocol was employed before docking such as removing duplicates, enumerating isomers, and generating 3D conformations. The protein-ligand docking active site was defined by the location of the original ligand. All other docking and consequent scoring parameters were kept at their default settings. The compound was considered to be a potentially active ingredient if the LibDock score was higher than the original ligand.

### 2.6. Network Construction and Analysis

Network construction was performed as follows:

1. The herbs, candidate compounds, and candidate targets of XFZYD were used to construct an herb-candidate compound-candidate target (HB-cC-cT) network.
2. The PPI data obtained above was used to establish the TBI-specific protein interaction network.
3. Herbs, potential compounds, and putative targets from XFZYD for treating TBI were used to build a herb-potential compounds-potential targets (HB-pC-pT) network.
4. Potential targets and the biology process they participate in were used to construct the pT-F network.
5. Compounds and targets through molecule docking validating were used to build a compound-target (C-T) network.

All networks were generated and analyzed by Cytoscape 3.4.0 [52], an open source of bioinformatic package for biological network analysis and visualization. Two topological parameters including degree and betweenness were calculated for the obtained networks which disclose the significance of a node.

### 2.7. Gene Ontology (GO) and Pathway Enrichment Analysis

The functional enrichment tool DAVID [53] (DAVID, https://david.ncifcrf.gov/), ver. 6.8) was used to calculate both the KEGG pathway and GO biological processes (BP) enrichment.

### 2.8. Statistical Analysis

All data were expressed as mean ± standard deviation (SD). The molecule descriptors data were analyzed by one-way ANOVA. The criterion for statistical significance was p < 0.05. Statistical analyses were conducted using the SPSS 24.0.

## 3. Results

TCM, an experience-based medicine, has been widely used for thousands of years. It has accumulated abundant clinical experience, forming a comprehensive and unique medical system [35]. The complexity of the phytochemical components makes it extremely difficult to illustrate the action mechanisms of XFZYD from a molecule or system level. As a chief mean of treating diseases clinically; generally TCM doctors prescribe formula based on the principle of “Jun-Chen-Zuo-Shi”: “Jun” (monarch) treats the main cause or primary symptoms of the disease. “Chen” (minister) enhances the actions of “Jun” or treats the accompanying symptoms. “Zuo” (adjuvant) not only reduces or eliminates the possible toxic effects of the Jun or Chen, but also treats the accompanying symptoms. “Shi” (guide) helps to deliver or guide the other herbs to the target organs [18]. According to the unique feature of TCM, our work tried to perform “Jun-Chen-Zuo-Shi” based system study to clarify the multiple mechanisms of XFZYD in the treatment of TBI.

### 3.1. Herbal Ingredient Comparison and Target Prediction of XFZYD

**Figure 2:**
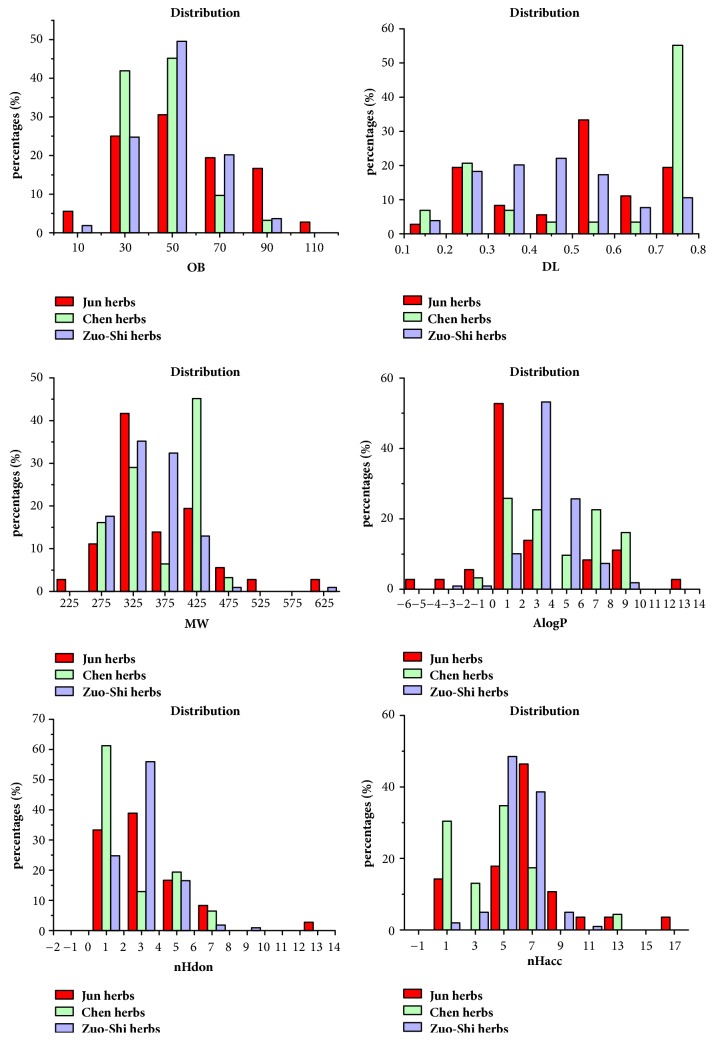
The profile distributions of six important molecular properties for all ingredients from the Jun, Chen, and Zuo-Shi herbs. OB, Oral bioavailability; MW, molecular weight; DL, drug-likeness; AlogP, partition coefficient between octanol and water; nHacc, number of hydrogen-bond acceptors; nHdon, number of hydrogen-bond donors.

**Figure 3:**
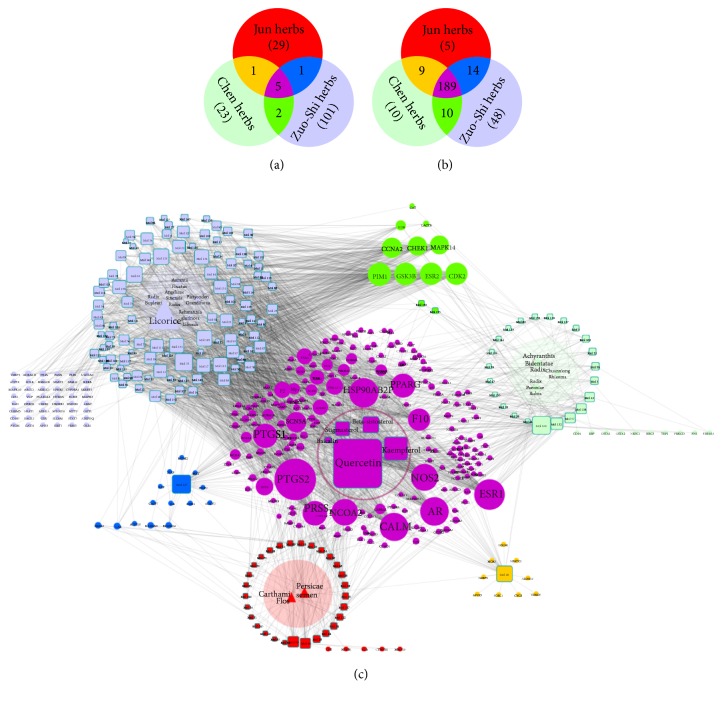
HB-cC-cT network of XFZYD. (a) and (b) The distribution of different candidate compounds and targets in the network (red: the Jun herbs-specific cC (a) and cT (b); aqua: the Chen herbs-specific cC (a) and cT (b); periwinkle: the Zuo-Shi herbs-specific cC (a) and cT (b); claybank: common cC (a) and cT (b) between the Jun and Chen herbs; blue: common cC (a) and cT (b) between the Jun and Zuo-Shi herbs; green: common cC (a) and cT (b) between the Chen and Zuo-Shi herbs; purple: common cC (a) and cT (b) among the 3 group of herbs. (c) The triangles with circle backgrounds represent the herbs (HB); the squares and circles represent the candidate compounds (cC) and candidate targets (cT). The red triangles, squares, and circles represent corresponding HB, cC, and cT in the Jun herbs; the same is to aqua representing the Chen herbs and periwinkle representing the Zuo-Shi herbs. The claybank squares and circles represent corresponding cC and cT overlap between the Jun and Chen herbs; the same is to blue representing the overlap between the Jun and Zuo-Shi herbs and the green representing the overlap between the Chen and Zuo-Shi herbs. The purple squares and circles represent the corresponding cC and cT shared by the 3 group of herbs. The size of the node is proportional to the value of degree.

We obtained 162 components originated from XFZYD. Of these compounds, 160 chemicals that were in accord with standard requirements were searched from the TCMSP database. The other 2 components, amygdalin and hydroxysafflor yellow A, were taken into consideration for their obvious pharmacological action as well. The detailed information of these compounds is showed in Table S1.* Persicae Semen (Tao ren)* and* Carthami Flos (Hong hua)*, the Jun (monarch) herbs of XFZYD, contained 36 bioactive components which accounted for 22% of the 162 chemicals.* Radix Paeoniae Rubra (Chi shao)*,* Chuanxiong Rhizoma (Chuan xiong)*, and* Achyranthis Bidentatae Radix (Niu xi)*, the Chen (minister) herbs, contained 31 bioactive components which accounted for 19% of the 162 chemicals.* Angelicae Sinensis* Radix* (Dang gui)*,* Rehmannia glutinosa Libosch (Sheng di huang)*,* Platycodon Grandiforus (Jie geng)*,* Aurantii Fructus (zhi ke)*,* Radix Bupleuri (chai hu)*, and* licorice (Gan cao)*, the Zuo-Shi (adjuvant and guide) herbs of XFZYD, contained 109 bioactive components which accounted for 67% of the 162 chemicals. Ingredients from these herbs were compared based on the 6 important drug-associated descriptors, including molecular weight (MW), number of hydrogen-bond donors (nHdon), number of hydrogen-bond acceptors (nHacc), partition coefficient between octanol and water (AlogP), oral bioavailability (OB), and drug-likeness (DL). The distributions of the 6 descriptors of the ingredients from the three groups are shown in Table 1 and Figure 2. We found no significant differences in the values of MW (p=0.16), nHdon (p=0.32), nHacc (p=0.61), and AlogP (p=0.82) among the 3 groups. However, the average OB value of compounds from the march herbs is 55.59±23.91, which was significantly different from the Chen herbs (OB=45.64±13.27, P=0.004) and the Zuo-Shi herbs (OB=48.77±15.05, P=0.021). The following 2 groups had no significant difference in OB value (P=0.272). As for DL, the Chen herbs revealed the highest DL index (0.53±0.23) which displayed significant difference from the Zuo-Shi herbs (0.44±0.19, p=0.012) while showing no difference with the Jun herbs (0.49±0.18 p=0.333).  Figure 3(a) indicated that 5 bioactive compounds were shared by the Jun, Chen, and Zuo-Shi herbs. One compound overlapped between the Jun and Chen herbs, while there were 2 compounds shared by the Chen and Zuo-Shi herbs. One compound overlapped between the Jun and Zuo-Shi herbs.

### 3.2. Target Prediction of XFZYD

A total of 285 potential targets from the 162 compounds were generated using the target prediction model. The amounts of potential targets hit by the Jun, Chen, and Zuo-Shi drugs were 217, 218, and 261, respectively. The detailed data of the targets is shown in Table S2. As depicted in Figure 3(b), there was a significant target overlap among the 3 groups (189 candidate targets), but less overlap between the Jun and Chen herbs (9 candidate targets). The number of targets shared by the Jun and Zuo-Shi herbs was 14, while 10 targets were overlapped between the Chen and Zuo-Shi herbs.

### 3.3. HB-cC-cT Network Construction and Analysis

We next established a HB-cC-cT network through network analysis to illuminate the relationship among the herbs, candidate compounds, and candidate targets (Figure 3(c)). This network consisted of 485 nodes (11 herbs, 162 candidate compounds, and 285 candidate targets) and 2585 edges. A herb (triangle) and cC (square) are connected if the compound is contained in this herb and the edges between cC and cT represent the interaction. The size of nodes is proportional to the value of degree. The larger size of the node means more pharmacologically important. Two centrality indicators, degree and betweenness, identify the important nodes within the network. Different centralities reflect different importance of nodes in a network from different angles. Interestingly, both of the two types of centrality indicators uniformly confirmed the most important 10 candidate compounds from XFZYD and the top 10 targets anchored by XFZYD (Tables 2 and 3). Figure 3(c) demonstrated that licorice possessed the largest degree (88) compared with other herbs originated from XFZYD. This implicated that it contained the most bioactive compounds (88), including quercetin (Mol 148, degree=153), kaempferol (Mol 108, degree=65), 7-Methoxy-2- methyl isoflavone (Mol 33, degree=44), formononetin (Mol 63, degree=39), naringenin (Mol 133, degree=39), isorhamnetin (Mol 105, degree=38), medicarpin (Mol 131, degree=35), and licochalcone a (Mol 113, degree=33), followed by* Persicae Semen* (degree=19),* Carthami Flos* (degree=18),* Achyranthis Bidentatae Radix* (degree=17),* Radix Paeoniae Rubra* (degree=14),* Radix Bupleuri *(degree=12),* Chuanxiong Rhizoma* (degree=6),* Aurantii Fructus* (degree=4),* Platycodon Grandiforus* (degree=4),* Rehmannia glutinosa Libosch* (degree=3), and* Angelicae Sinensis Radix *(degree=2).* Persicae Semen *(degree=19) and* Carthami Flos *(degree=18), the Jun herbs from XFZYD, possessed 29 specific compounds and 5 unique target proteins including ALB, CTNNB1, MMP10, LYZ, and NFKB1. Twenty-three Chen-specific potential compounds targeted 10 specific proteins including CD14, LBP, NR3C1, BBC3, TEP1, PRKCD, FN1, PDE10A, GSTA1, and GSTA2. The Zuo-Shi herbs possessed the largest number of specific compounds (101) and 48 unique proteins such as HTR3A, ADRA1D, PYGM, OLR1, CHRM5, RXRB, STAT3, MAPK10, OPRD1, and MAPK3. There were 189 proteins anchored by the Jun, Chen, and Zuo-Shi drugs.

Among the 162 candidate compounds, quercetin had the largest value of degree (153), implicating its critical role in XFZYD. The four herbs, namely,* Carthami Flos *(Jun),* Achyranthis Bidentatae Radix* (Chen), and* licorice, *and* Radix Bupleuri* (Zuo-Shi) contained quercetin. It targeted 149 bioactive proteins. PTGS2 (degree=126), HSP90AB2P (degree=85), AR (degree=81), NCOA2 (degree=75), PRSS1 (degree=68), PTGS1 (degree=67), PPARG (degree=66), and F10 (degree=60) were predicted as the major candidate targets of quercetin, followed by kaempferol (degree=65), which was contained by* Carthami Flos* (Jun),* Achyranthis Bidentatae Radix *(Chen), and* Radix Bupleuri, licorice *(Zuo-Shi). It targeted 61 bioactive proteins including PTGS2 (degree=126), HSP90AB2P (degree=85), CALM (degree=81), AR (degree=81), NOS2 (degree=76), and NCOA2 (degree=75). Quercetin, stigmasterol, kaempferol, baicalin, and beta-sitosterol existed in 3, the Jun, Chen, and Zuo-Shi, drugs, demonstrating crucial roles of these components. The 5 ingredients in the Jun, Chen, and Zuo-Shi herbs targeted 186 bioactive proteins which accounted for 65% targets of XFZYD. Baicalein existed in the Jun and Chen herbs. Luteolin existed in the Jun and Zuo-Shi herbs. Spinasterol and sitosterol existed in the Chen and Zuo-Shi herbs. 126 bioactive compounds targeted PTGS2, followed by ESR1 (88), HSP90AB2P (85), AR (81), CALM (81), NOS2 (76), NCOA2 (75), PRSS1 (68), PTGS1 (67), and PPARG (66). As depicted in Figure 3(c), these target proteins were anchored by ingredients in the 3 group drugs.

The analysis of the network revealed that quercetin (Mol 148), kaempferol (Mol 108), luteolin (Mol 127), wogonin (Mol 160), 7-Methoxy-2-methyl (Mol 33), and beta-sitosterol (Mol 41) were predicted as the major active compounds of XFZYD. The proteins including F2, NOS2, PTGS1, PTGS2, and CALM were predicted as essential pharmacological proteins for the therapeutic effects of XFZYD.

### 3.4. Analyses on TBI Based Specific Protein Interaction Network

**Figure 4:**
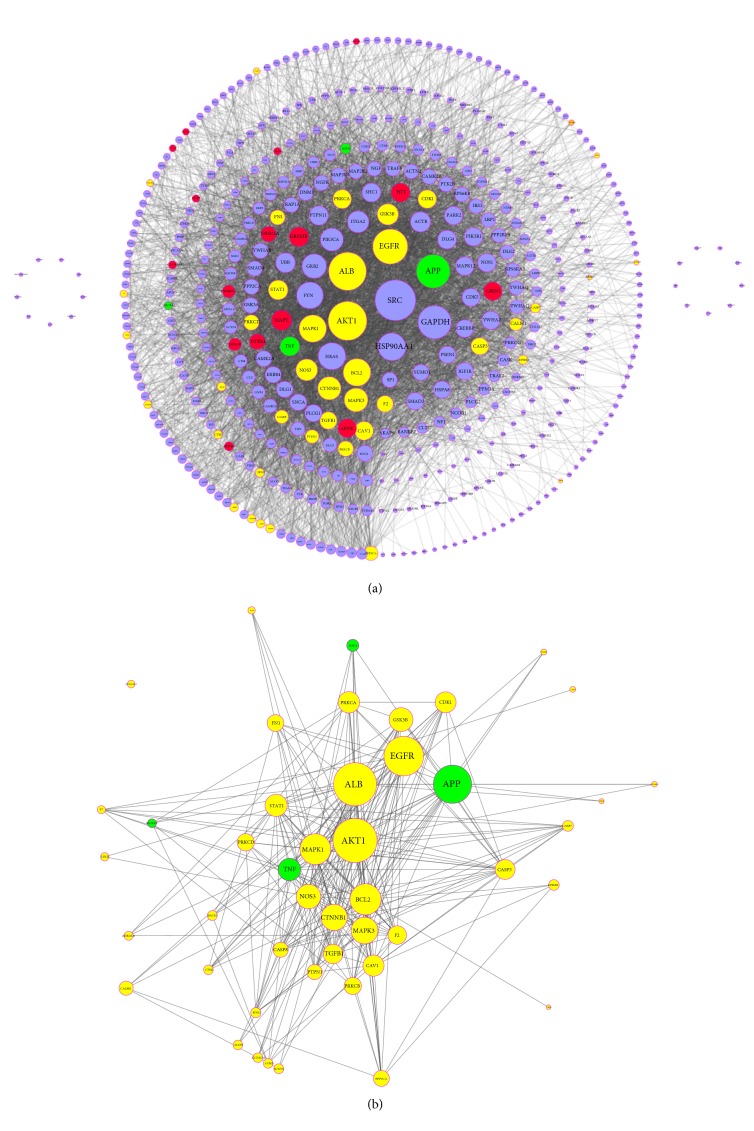
TBI-related protein interaction network. (a) 21 hub proteins (red and green) were identified through the analysis of Therapeutic Target Database (TTD), as well as Online Mendelian Inheritance in Man (OMIM). 4 overlapped protein targets (green) were obtained between 21 hub proteins in TBI and candidate targets of XFZYD. Periwinkle: proteins from HPRD and STRING analyses were not targeted by XFZYD. (b) 47 candidate protein targets of XFZYD were screened for treating TBI. Yellow: candidate protein targets of XFZYD. The size of nodes is proportional to the value of degree.

**Figure 5:**
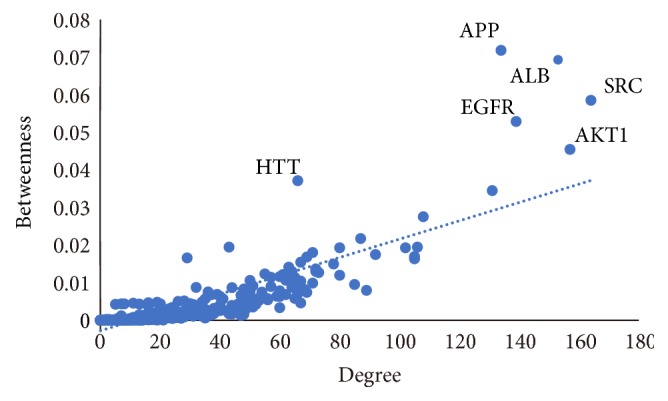
Relationship between degree and betweenness centrality in the TBI-specific protein interaction network.

Network biology integrated with different kinds of data, including physical or functional networks and disease gene sets, is used to interpret human diseases. Protein-protein interaction networks (PPI) are fundamental to understand the cellular organizations, biological processes, and protein functions [54]. From the systematic perspective, the analysis of TBI-related PPI will improve the understanding of the complicated molecular pathways and the dynamic processes underlying TBI. We screened the TBI-specific genes and protein targets using Online Mendelian Inheritance in Man database (OMIM) and Therapeutic Target Database (TTD).  Figure 4 indicated that 21 TBI-specific genes/proteins were acquired. Further these hub-proteins were submitted to HPRD and STRING to establish the TBI-specific protein interaction network. The detailed information of the TBI-specific proteins is shown in Table S3. The results suggested that the network consisted of 489 nodes and 5738 edges (Figure 4(a)). We obtained top 10 TBI-related proteins according to 2 centrality indicators generated and summarized in Table 4. Interestingly, we found that the node with higher betweenness tends to possess larger degree (Figure 5). Network topology analysis showed that protooncogene tyrosine-protein kinase Src (SRC, degree=164, betweenness=0.059), RAC-alpha serine/threonine-protein kinase (AKT1, degree=157, betweenness=0.045), Serum albumin (ALB, degree=153, betweenness=0.069), Epidermal growth factor receptor (EGFR, degree=139, betweenness=0.053), and Amyloid beta A4 protein (APP, degree=134, betweenness=0.072) contributed to the essential role in the pathophysiology of TBI. All of these indicated that the top mutual target proteins performed various beneficial functions to treat TBI at the molecular level. For example, SRC is activated following engagement of many different classes of cellular receptors. It participates in signal pathways that control a diverse spectrum of biological activities including gene transcription, immune response, cell adhesion, cell cycle progression, apoptosis, migration, and transformation [55]. SRC can result in blood-brain barrier (BBB) disruption and brain edema at the acute stage; the inhibition of SRC family kinases can protect hippocampal neurons and improve cognitive function after TBI [56]. AKT1 regulates many processes including metabolism, proliferation, cell survival, growth, and angiogenesis [57]. The PI3K/AKT/PTEN pathway has been shown to play a pivotal role in neuroprotection, enhancing cell survival by stimulating cell proliferation and inhibiting apoptosis after TBI [58]. ALB is the main protein of plasma and can be a biomarker to predict outcome of TBI [59]. Its main function is the regulation of the colloidal osmotic pressure of blood. We found that it may participate in pathological process of TBI.

KEGG pathway analysis was also used to determine the functions of proteins.  Table 5 describes the top 10 significantly enriched KEGG pathways. These pathways play crucial roles in pathophysiology process which have also been widely discussed in existing literature. For instance, MAPK signaling pathway can promote pathological axonal death through triggering a local energy deficit [60]. Neurotrophin signaling through Trk receptors regulates cell survival, proliferation, the fate of neural precursors, axon, and dendrite growth and patterning, and the expression and activity of functionally important proteins, such as ion channels and neurotransmitter receptors [61]. Engagement of cells with the extracellular matrix (ECM) proteins is crucial for various biological processes, including cell adhesion, differentiation, and apoptosis, contributing to maintenance of tissue integrity and wound healing [62]. The 47 TBI-specific proteins targeted by XFZYD (yellow and green) were further discussed below.

### 3.5. HB-pC-pT Network, pT-F Network Construction, and Molecule Docking Analyses of XFZYD for the Treatment of TBI

**Figure 6:**
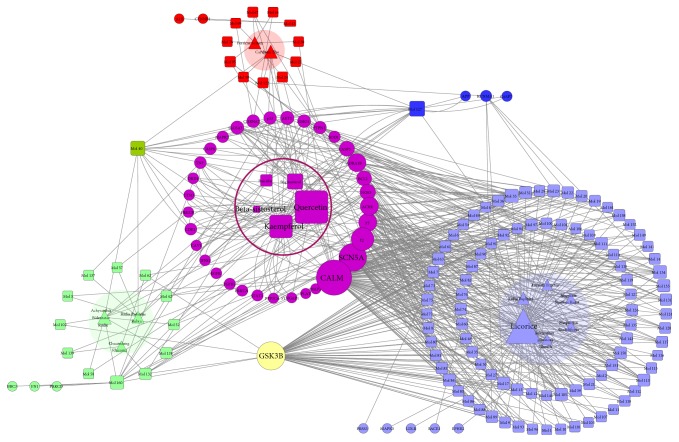
HB-pC-pT network of XFZYD for treating TBI. The triangles with circle backgrounds represent the herbs (HB); the squares and circles represent the potential compounds (pC) and targets (pT). The red triangles, squares, and circles represent corresponding HB, pC, and pT in the Jun herbs; the same is to aqua representing the Chen herbs and periwinkle representing the Zuo-Shi herbs. The claybank squares and circles represent corresponding pC and pT shared by the Chen and Zuo-Shi herbs; the same is to blue representing the overlap between the Jun and Zuo-Shi herbs and the green representing the overlap between the Chen and Zuo-Shi herbs. The purple squares and circles represent the corresponding pC and pT shared by the 3 groups of herbs.

To investigate the therapeutic mechanisms of XFZYD for the treatment of TBI, a HB-pC-pT network of XFZYD for treating TBI was built (Figure 6). 47 TBI-specific target proteins (circles) were targeted by 119 potential compounds (squares) from XFZYD (Figure 6). Detailed information for the 119 potential compounds is shown in Table S4. Similarly, 5 pharmacologically active ingredients in the Jun, Chen, and Zuo-Shi groups, including quercetin, stigmasterol, kaempferol, baicalin, and beta-sitosterol, anchored 33 TBI-specific proteins such as CALM, SCN5A, F2, ACHE, F7, ADRA1B, NOS3, BCL2, CASP3, and AKT1. 11 Jun-specific compounds targeted 2 specific targets (ALB, CTNNB1), while 12 Chen-specific compounds anchored 3 unique proteins (PRKCD, FN1, and BC3). The Zuo-Shi herbs possessed the largest number of active compounds (89) and targeted 5 unique proteins including EPHB2, BACE1, LDLR, MAPK3, and PRSS3. GSK3B was the common target between the Chen and Zuo-Shi drugs. KCNMA1, CASP7, and APP were targeted by the Jun and Zuo-Shi drugs.

The top 10 candidate compounds and targets to treat TBI were showed in Tables 6 and 7. For most of active compounds from XFZYD, each component hit more than one target.  Table 6 demonstrated that quercetin had the highest number of targets (degree =76), followed by kaempferol (degree =43), beta-sitosterol (degree =35), stigmasterol (degree =19), luteolin (degree=18), baicalein (degree=15), 7-Methoxy-2-methyl isoflavone (degree=12), wogonin (degree=12), nobiletin (degree=11), and naringenin (degree=9). For instance, quercetin (3,3′,4′,5,7-pentahydroxyflavone) is a naturally occurring flavonoid commonly found in fruits and vegetables. It regulates multiple biological pathways eliciting induction of apoptosis as well as inhibiting angiogenesis and proliferation [63, 64]. Quercetin can attenuate neuronal autophagy and apoptosis in rat traumatic brain injury model via activation of PI3K/Akt signaling pathway [65]. It has also been reported to have a protective ability against oxidative stress and mutagenesis in normal cells [66, 67]. Kaempferol (3, 4′, 5, 7 tetrahydroxy flavone) is a yellow-colored flavonoid that is widely distributed in many botanical families [68]. It has been shown to possess a variety of biological characteristics, including effects of anti-inflammatory [69], antioxidative [70], tumor growth inhibition [71], and alleviating insulin resistance in type 2 diabetic rats [72]. Beta-sitosterol (BS) is a vegetable-derived compound found in various plants and is suggested to modulate the immune function, inflammation, and pain levels by controlling the production of inflammatory cytokines [73].

For targets analysis, CALM possesses the largest degree (degree =84), followed by GSK3B (degree =61), SCN5A (degree = 59), F2 (degree = 43), ACHE (degree=28), F7 (degree=28), ADRA1B (degree=28), NOS3 (degree=20), BCL2 (degree=18), and CASP3 (degree=18), which demonstrated their crucial therapeutic effects for treating TBI. For instance, CALM possess an essential position in calcium signaling pathway and is related to morphological changes, migration, proliferation, and secretion of cytokines and reactive oxygen species of Microglial cells [74]. The activation of CaMKII*α*, major isoform of Ca^2+^/calmodulin-dependent protein kinase (CaMK) in brain, is directly associated with the production of proinflammatory cytokines, such as TNF-*α* and IL-1*β* [75]. GSK-3*β* is a serine/threonine-protein kinase, which is abundant in the central nervous system (CNS), particularly in neurons [76]. It can control gene transcription, axonal transport, and cytoskeletal dynamics in growth cones [77]. The inhibition of GSK-3*β* attenuates apoptotic signals and prevents neuronal death [78]. There is increasing evidence that prothrombin (F2) and its active derivative thrombin are expressed locally in the central nervous system. Beside the central role in the coagulation cascade, the generation of thrombin leads to receptor mediated inflammatory responses, cell proliferation/modulation, cell protection, and apoptosis [79, 80]. The role in brain injury depends upon its concentration, as higher amounts cause neuroinflammation and apoptosis, while lower concentrations might even be cytoprotective [81].

**Figure 7:**
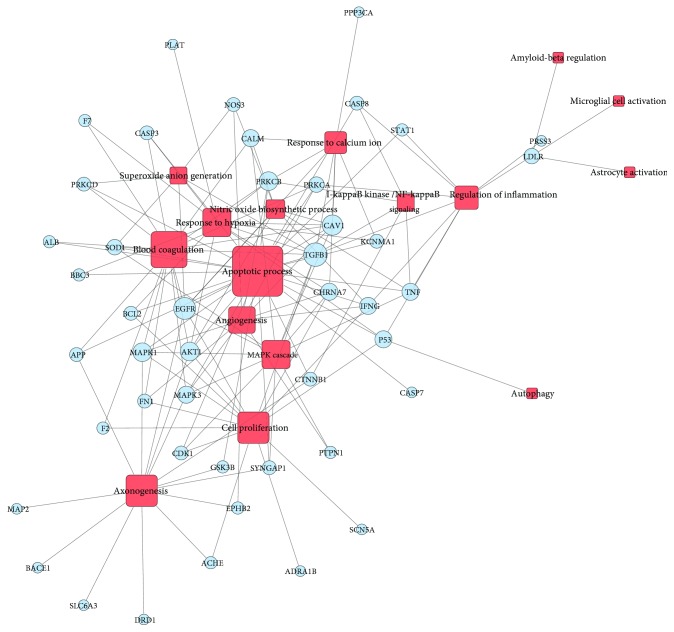
pT-F network of XFZYD for treating TBI. 16 biological processes (red square) the 47 target proteins (periwinkle circle) of XFZYD participate in for treating TBI.

Direct tissue damage, as well as hypoxic-ischemic increasing anaerobic glycolysis of the brain tissue, results in the ATP-stores depletion and failure of energy-dependent membrane ion pumps, especially for voltage-dependent Ca^2+^ and Na^+^-channels. Accumulated Ca^2+^ activates lipid peroxidases, proteases, and phospholipases and caspases at the same time, increasing the intracellular concentration of free fatty acids and free radicals, leading to necrosis or apoptosis of neurocyte [82]. At the same time, the activation of resident glial cells, microglia, and astrocytes and the infiltration of blood leukocytes secrete various immune mediators elicited inflammatory responses, which subsequently intersect with adjacent pathological cascades including oxidative stress, excitotoxicity, or reparative events including angiogenesis, scarring, and neurogenesis [83]. Function analysis of the 47 target proteins regulated by XFZYD was mainly associated with core pathophysiology process of TBI (Figure 7). 47 target proteins were connected with 16 key process related to TBI. Most of the targets have one or more links to other biological process such as apoptosis, cell proliferation, superoxide anion generation, nitric oxide biosynthetic process, response to calcium ion, I-kappaB kinase/NF-kappaB signaling, and regulation of inflammation. The above analysis implied the multifunction character of these target proteins regulated by XFZYD. Of these target proteins, 25 proteins (53% of the 47), such as TGFB1, EGFR, CAV1, MAPK1, PRKCB, and AKT1 were responsible for regulating the apoptosis process, 17 for blood coagulation, 14 for cell proliferation and axon genesis, and 12 for hypoxia and MAPK cascade. We found that TGFB1 was the crucial protein, because it participated in 9 biological processes related to TBI such as apoptosis, blood coagulation, cell proliferation, and MAPK cascade followed by EGFR (8), CAV1 (7), MAPK1 (6), PRKCB (6), and AKT1 (6).

Overall, these observations strongly support the evidence that the generated HB-pC−pT network and pT-F network have important roles in treating TBI, further validating the drug targeting approach.

**Figure 8:**
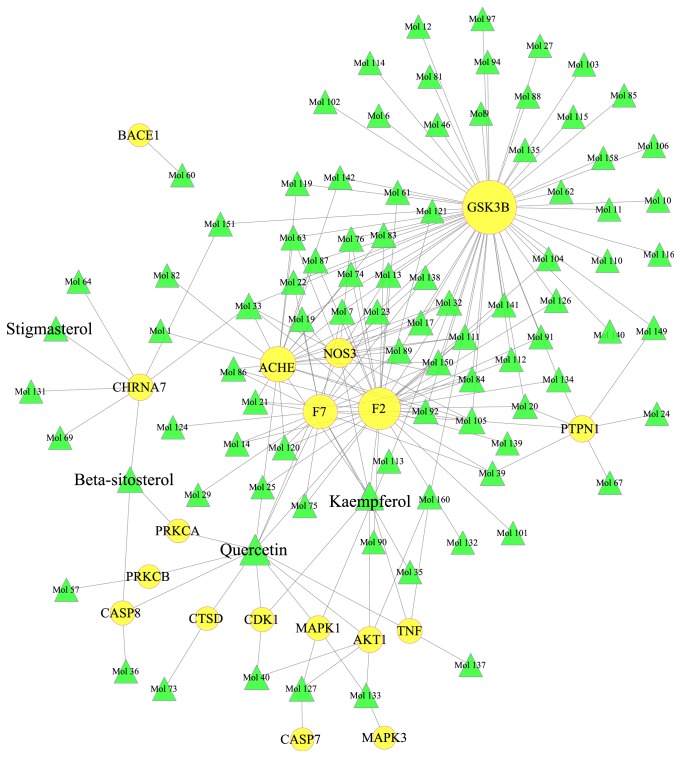
C-T network through molecule docking validating. 119 potential compounds (green triangles) interacting with 18 potential targets (yellow circles) of XFZYD. The size of the nodes is proportional to the value of degree.

**Figure 9:**
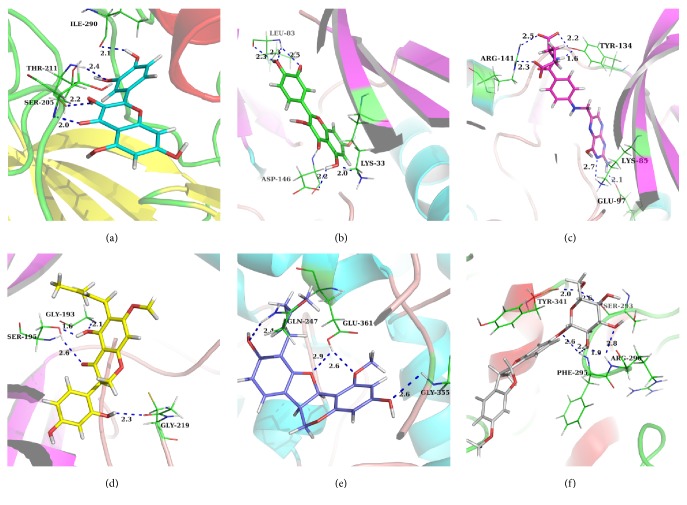
Hydrogen-bonding networks within the binding site of the compound−target complexes obtained from molecule docking. (a) AKT1-quercetin, (b) CDK1-quercetin, (c) GSK3B-FA, (d) F2-glyasperin B, (e) NOS3-1-Methoxyphaseollidin, and (f) ACHE-(-)-Medicocarpin. The molecules are presented as ball and stick models. Active site amino acid residues are represented as lines. Dotted blue lines in these pictures represent hydrogen bonds with distance unit of Å. Other O and N atoms are colored as red and blue, respectively.

Molecule docking was used to further validate the binding mode between candidate compounds and their target proteins. We found that 18 TBI-specific target proteins interacted with 91 candidate compounds from XFZYD (Figure 8). Other 29 target proteins were not discussed for the lack of proper protein crystal structure. The detailed molecule docking results are shown in Table S5. The 6 essential proteins including GSK3B, AKT1, CDK1, F2, NOS3, and ACHE were used to elucidate the exact binding mode (Figure 9). Quercetin was located within the binding cavity of AKT1 and CDK1 (Figures 9(a) and 9(b) and S1A, B). Four conventional hydrogen bonds were formed between quercetin and AKT1 by interacting with the key amino acids including ILE-290, THR-211, and SER 205. Additionally, *π*-*π* interactions between quercetin and TRP-90 were found in the active site which helped the stabilization of the compound at the binding site (Figure 9(a), S1A).  Figure 9(b) and S1B suggested that five conventional hydrogen bonds (LEU-83, ASP-146, and LYS-33) and *π*-*π* interaction (PHE-80) were formed between quercetin and CDK1. The GSK3B-FA complexes (Figure 9(c), S1C) were stabilized by 6 hydrogen-bonding interactions between FA and LYS-85, GLU-97, TYR-134, and ARG-141. Glyasperin B mainly bonds to F2 through hydrogen bonds by interacting with the key amino acids including GLY-193, SER-195, and GLY-219 (Figure 9(d), S1D), and an edge-to-face *π*−*π* interaction was also observed with TYR-228. The GLN-247, GLU-351, and GLY-355 from the active site pocket of NOS3 participated in the hydrogen-bond formation with 1-Methoxyphaseollidin (Figure 9(e), S1E). The (-)-Medicocarpin formed a total of 6 hydrogen bonds with SER-293, PHE-295, ARG-296, and TRY-341 in the active site of ACHE (Figure 9(f), S1F). Besides, an edge-to-face *π*−*π* interaction was also observed with TYR-337. From the results, hydrogen-bonding and edge-to-face *π*−*π* interactions play key roles in the protein−ligand recognition and stability, which may be helpful in determining the inhibitor activities. And the C-T network confirmed the potential therapeutic effects of the candidate compounds from XFZYD to treat TBI through interacting with the relevant proteins. The computational analysis further elucidated the accurate molecule mechanisms between active compounds and targets.

## 4. Discussion

Traumatic brain injury (TBI) is a growing public health problem worldwide and is a leading cause of death and disability [84]. Although major progress has been made in understanding the pathophysiology of this injury, this has not yet led to substantial improvements in outcome by a lack of treatments which have proven successful during phase III trials for modern medicine [85, 86]. TCM, rooted in thousands of years of history, may offer an alternative or a complementary strategy for the treatment of TBI. XFZYD, a representative formula in TCM, has been used for years to treat TBI in China and has been demonstrated to be effective in clinical practice. However, its “multicomponents” and “multitargets” features make it much difficult to decipher the molecular mechanisms of XFZYD in the treatment of TBI from a systematic perspective if employing routine methods.

In the present study, a network pharmacology-based method was employed to elucidate the pharmacological mechanisms of XFZYD to treat TBI according to the drug combination principle of TCM. We first proposed a new modeling system, combining OB and DL screening, multiple drug targets prediction and validation, network construction, and molecule docking, to probe the efficiency of a typical TCM formula XFZYD for the treatment of TBI. The 11 herbs from XFZYD possessed 162 bioactive compounds and targeted 285 proteins. There were 5 compounds and 189 target proteins overlapped among the Jun, Chen, and Zuo-Shi group. Furthermore, 47 TBI-specific proteins were targeted by 119 (73%) bioactive compounds from XFZYD. Similarly, 5 common compounds and 33 (70%) common target proteins among the 3 groups of drugs were observed. Most of the bioactive ingredients targeted more than one protein. The 47 target proteins regulated several essential pathophysiological processes of TBI that referred to apoptosis, inflammation, cell proliferation, superoxide anion generation, nitric oxide biosynthetic process, response to calcium ion, etc. The above analysis reveals that the synergistic action mechanisms of XFZYD may be (1) bioactive compounds overlapping among the different group of herbs; (2) specific bioactive compounds from different groups of herbs targeting the same proteins; (3) specific bioactive compounds from different groups of herbs targeting different proteins which participate in the same pathophysiological process of the disease. To a certain degree, the 5 compounds including quercetin, stigmasterol, kaempferol, baicalin, and beta-sitosterol played essential role in XFZYD for TBI treatment. MAPK3, MAPK1, AKT1, PRKCA, TNF, PRKCB, EGFR, BCL2, GSK3B, CASP3, PPP3CA, and NOS3 were the main target proteins regulated by XFZYD in the treatment of TBI.

Interestingly, Beta-carotene (Mol 43) from* Carthami Flos* (the Jun herb) specifically regulated *β*-Catenin (CTNNB1) and played critical role for curing TBI. The *β*-Catenin is a critical downstream component of the Wnt pathway, which plays essential role in the regulation of mammalian neural development [87]. In vitro and in vivo studies demonstrate that the Wnt/*β*-catenin pathway regulates the proliferation and differentiation of neural progenitor cells [88]. Neuronal differentiation is induced by overexpression of *β*-catenin or the pharmacological inhibition of GSK3*β* (the phosphorylating enzyme of *β*-catenin) [89, 90]. This pathway also promotes blood vessel formation during vascular development, as well as the vascular repair process after TBI [91]. In addition, wogonin (Mol 160) from* Achyranthis Bidentatae Radix* (the Chen herb) specifically targeted fibronectin (FN1), Bcl-2-binding component 3 (BBC3), and Protein Kinase C delta type (PRKCD). FN1, an important component of the extracellular matrix (ECM) environment, promotes cell migration, neurite outgrowth, and synapse formation during neural development [92]. It aggregates in the injured brain and plays a neuroprotection role through antiapoptosis and anti-inflammation ways following TBI [93, 94]. BBC3, namely p53 upregulated modulator of apoptosis (PUMA), is critical for the p53-dependent apoptosis pathway which plays an important role in hippocampal neuronal loss and associated cognitive deficits [95]. PRKCD, one of PKC isoforms, activates signal transduction pathways involved in neuronal regeneration [96], synaptic transmission/plasticity [97], and activation of apoptosis processes [98] as well as higher brain functions such as learning and memory [99]. The activators of PKC are effective for the treatment of TBI [100]. Furthermore, Mitogen-activated protein kinase 3 (MAPK3), Beta-secretase 1 (BACE1), Ephrin type-B receptor 2 (EphB), and low-density lipoprotein receptor (LDLR) were specifically targeted by the ingredients in the Zuo-Shi herbs. Naringenin (Mol 133) targeted LDLR, MAPK3, while euchrenone (Mol 60) anchored BACE1 and nobiletin (Mol 134) targeted EphB2. MAPK3 is an essential component of the MAP kinase signal transduction pathway. It has been appreciated recently that the ERK1/2 cascade plays a fundamental role in synaptic plasticity and memory [101]. BACE1 is responsible for production of A*β* from amyloid precursor protein (APP). A*β* can cause cell death, activate inflammatory pathways [102], and prime proapoptotic pathways for activation by other insults [103]. The blocking of BACE1 can ameliorate motor and cognitive deficits and reduce cell loss after experimental TBI in mice [104]. EphB is localized to synaptic sites in hippocampal neurons [105]. The interaction between EphB and NMDA receptors regulates excitatory synapse formation [106]. LDLR acts as an important receptor that facilitates brain A*β* clearance and inhibits amyloid deposition [107] and then ameliorates Alzheimer's disease neuropathology after TBI [108]. The analysis above indicates the “Jun”, “Chen”, and “Zuo-Shi” herbs from XFZYD trigger their specific targets regulation, respectively, for the therapeutic effects.

XFZYD is a very famous traditional Chinese formula in promoting qi circulation and removing blood stasis according to TCM theory. However, several limited researches have demonstrated its efficacy for treating TBI, such as anti-inflammatory and synaptic regulation [30, 31], which are in accord with our study. However, previous studies merely partially deciphered the molecule mechanism of XFZYD for treating TBI. This study reports 119 bioactive compounds in XFZYD that target 47 TBI-specific proteins such as MAPK3, MAPK1, AKT1, PRKCA, TNF, PRKCB, and EGFR. These proteins regulate several crucial pathophysiological processes of TBI, such as apoptosis, inflammation, blood coagulation, and axon genesis. Our study demonstrates that the therapeutic actions of XFZYD refer to “multicompounds”, “multitargets” features, rather than only the improvement of blood circulation. With the help of molecule docking method, we further validate the interactions between bioactive compounds and potential targets of XFZYD. The hydrogen-bonding and edge-to-face *π*−*π* interactions play key roles in the protein−ligand recognition and stability. This provides a valuable reference for further experimental investigations of bioactive ingredients and therapeutic targets of XFZYD for treating TBI.

## 5. Conclusion

Our work successfully illuminates the efficiency of XFZYD for the treatment of TBI, as well as herb combination rule of TCM formula. Network pharmacology with molecule docking method confirms the “multicompounds, multitargets” therapeutic actions of XFZYD in the treatment of TBI. The present work may provide valuable evidence for further clinical application of XFZYD for treating TBI.

## Figures and Tables

**Table 1:** Comparison of molecular properties among the Jun, Chen, and Zuo-Shi herbs.

INDEX	MW	nHdon	nHacc	AlogP	OB	DL
	(mean ± SD)	(mean ± SD)	(mean ± SD)	(mean ± SD)	(mean ± SD)	(mean ± SD)
Jun herbs	380.28 (94.23)	2.65 (2.26)	5.11 (3.21)	3.37 (4.36)	55.59 (23.91)	0.49 (0.18)
Chen herbs	381.80 (91.60)	2.18 (1.95)	5.5 (3.47)	3.14 (3.15)	45.64 (13.27)	0.53 (0.23)
Zuo-shi herbs	357.45 (88.22)	2.61 (1.60)	5.6 (2.49)	3.44 (1.85)	48.77 (15.05)	0.44 (0.19)

OB, oral bioavailability; MW, molecular weight; DL, drug-likeness; AlogP, partition coefficient between octanol and water; nHacc, number of hydrogen-bond acceptors; nHdon, number of hydrogen-bond donors.

**Table 2:** Top 10 candidate compounds according to 2 centrality indicators.

**Compounds**	**Degree**	**Compounds**	**Betweenness**
quercetin	153	quercetin	0.35875838
kaempferol	65	naringenin	0.08768253
luteolin	48	kaempferol	0.07665228
wogonin	46	luteolin	0.05736204
7-Methoxy-2-methyl isoflavone	44	baicalein	0.05542898
beta-sitosterol	41	beta-sitosterol	0.041007
baicalein	40	wogonin	0.03968963
formononetin	39	Stigmasterol	0.03586788
naringenin	39	nobiletin	0.03057461
isorhamnetin	38	formononetin	0.03051437

**Table 3:** Top 10 target proteins of XFZYD according to 2 centrality indicators.

**Proteins**	**Degree**	**Proteins**	**Betweenness**
PTGS2	126	PTGS2	0.128936
ESR1	88	NCOA2	0.060806
HSP90AB2P	85	HSP90AB2P	0.049647
AR	81	PRKACA	0.046484
CALM	81	PTGS1	0.04365
NOS2	76	AR	0.030252
NCOA2	75	PRSS1	0.025575
PRSS1	68	ESR1	0.022212
PTGS1	67	PPARG	0.021403
PPARG	66	PGR	0.020685

Note: the centrality indicators identify the important nodes within the network. Higher degree centrality and betweenness centrality indicate greater importance.

**Table 4:** Top 10 proteins of TBI specific proteins according to 2 centrality indicators.

**Proteins**	**Degree**	**Proteins**	**Betweenness**
SRC	164	APP	0.071814
AKT1	157	ALB	0.069343
ALB	153	SRC	0.058568
EGFR	139	EGFR	0.052938
APP	134	AKT1	0.045466
GAPDH	131	HTT	0.037164
HSP90AA1	108	GAPDH	0.034549
BCL2	106	HSP90AA1	0.027596
MAPK1	105	CTNNB1	0.021759
HRAS	105	GNB1	0.019492

Note: the centrality indicators identify the important nodes within the network. Higher degree centrality and betweenness centrality indicate greater importance.

**Table 5:** Top 10 significantly enriched KEGG pathways in TBI-specific proteins.

**Pathway ID**	**Pathway description**	**Gene count**	**FDR**
4010	MAPK signaling pathway	44	2.99E-23
5200	Pathways in cancer	43	1.13E-18
4722	Neurotrophin signaling pathway	41	1.01E-34
4151	PI3K-Akt signaling pathway	40	1.11E-15
4510	Focal adhesion	39	2.92E-22
5205	Proteoglycans in cancer	39	4.10E-21
5010	Alzheimer s disease	34	1.24E-20
4015	Rap1 signaling pathway	33	7.69E-17
4014	Ras signaling pathway	33	6.46E-16
4020	Calcium signaling pathway	31	5.05E-17

**Table 6:** Top 10 potential candidate compounds of XFZYD for treating TBI according to 2 centrality indicators.

**Compound**	**Degree**	**Compound**	**Betweenness**
quercetin	76	quercetin	0.1682179
kaempferol	43	kaempferol	0.05501511
beta-sitosterol	35	wogonin	0.05141376
Stigmasterol	19	beta-sitosterol	0.04451294
luteolin	18	naringenin	0.03251738
baicalein	15	beta-carotene	0.0297721
7-Methoxy-2-methyl isoflavone	12	nobiletin	0.02787992
wogonin	12	7-Methoxy-2-methyl isoflavone	0.02096439
nobiletin	11	luteolin	0.02090223
naringenin	9	baicalein	0.01453488

**Table 7:** Top 10 potential targets of XFZYD for treating TBI according to 2 centrality indicators.

**Targets**	**Degree**	**Targets**	**Betweenness**
CALM	84	CALM	0.21452046
GSK3B	61	SCN5A	0.11441591
SCN5A	59	GSK3B	0.09553896
F2	43	F2	0.0689171
ACHE	28	BCL2	0.02651903
F7	28	CASP3	0.02372836
ADRA1B	28	F7	0.02032647
NOS3	20	ACHE	0.01845996
BCL2	18	ADRA1B	0.01704505
CASP3	18	NOS3	0.0166699

## Data Availability

The data used to support the findings of this study are available from the corresponding author upon request.

## Abbreviations

TBI: Traumatic brain injury
XFZYD: Xuefu Zhuyu decoction
TCM: Traditional Chines medicine
DL: Drug-likeness
OB: Oral bioavailability
HB-cC-cT: Herb-candidate compound-candidate target
HB-pC-pT: Herb-potential compound-potential target
CALM: Calmodulin
GSK3B: Glycogen synthase kinase-3 beta
SCN5A: Sodium channel protein type 5 subunit alpha
F2: Prothrombin
ACHE: Acetylcholinesterase
GO: Gene Ontology
KEGG: Kyoto Encyclopedia of Genes and Genomes.

## Supplementary Materials

Supplementary Materials Table S1: 162 bioactive compounds in XFZYD. Table S2: target proteins of XFZYD. Table S3: TBI-specific proteins. Table S4: 119 potential compounds of XFZYD for treating TBI. Table S5: docking result of 18 target proteins with 91 potential compounds. Fig. S1: the exact binding mode between active ingredients and protein targets obtained from molecule docking. (A) AKT1-quercetin, (B) CDK1-quercetin, (C) GSK3B-FA, (D) F2-glyasperin B, (E) NOS3-1-Methoxyphaseollidin, and (F)ACHE-(-)-Medicocarpin.
