# Supplementary material for: Exploring Pharmacological Mechanisms of Xuefu Zhuyu Decoction in the Treatment of Traumatic Brain Injury via a Network Pharmacology Approach
Source: Evid Based Complement Alternat Med. 2018 Oct 4;2018:8916938. doi: 10.1155/2018/8916938 (PMC6193325; doi:10.1155/2018/8916938)
Supplement: Supplementary Materials — Table S1: 162 bioactive compounds in XFZYD. Table S2: target proteins of XFZYD. Table S3: TBI-specific proteins. Table S4: 119 potential compounds of XFZYD for treating TBI. Table S5: docking result of 18 target proteins with 91 potential compounds. Fig. S1: the exact binding mode between active ingredients and protein targets obtained from molecule docking. (A) AKT1-quercetin, (B) CDK1-quercetin, (C) GSK3B-FA, (D) F2-glyasperin B, (E) NOS3-1-Methoxyphaseollidin, and (F)ACHE-(-)-Medicocarpin. [file 8916938.f1.docx]

**Supplementary materials**

**Table S1.** 162 bioactive compounds in XFZYD

| **Molecule Name** | **Mol ID** | **OB (%)** | **DL** | **database** |
| --- | --- | --- | --- | --- |
| hederagenin | MOL000296 | 36.91 | 0.75 | TcmSP |
| beta-sitosterol | MOL000358 | 36.91 | 0.75 | TcmSP |
| campesterol | MOL000493 | 37.58 | 0.71 | TcmSP |
| Sitosterol alpha1 | MOL001323 | 43.28 | 0.78 | TcmSP |
| 2,3-didehydro GA70 | MOL001328 | 63.29 | 0.5 | TcmSP |
| 2,3-didehydro GA77 | MOL001329 | 88.08 | 0.53 | TcmSP |
| GA120 | MOL001340 | 84.85 | 0.45 | TcmSP |
| GA121-isolactone | MOL001342 | 72.7 | 0.54 | TcmSP |
| GA122-isolactone | MOL001344 | 88.11 | 0.54 | TcmSP |
| 4a-formyl-7alpha-hydroxy-1-methyl-8-methylidene-4aalpha,4bbeta-gibbane-1alpha,10beta-dicarboxylic acid | MOL001349 | 88.6 | 0.46 | TcmSP |
| Gibberellin A44 | MOL001351 | 101.61 | 0.54 | TcmSP |
| GA54 | MOL001352 | 64.21 | 0.53 | TcmSP |
| GA60 | MOL001353 | 93.17 | 0.53 | TcmSP |
| GA63 | MOL001355 | 65.54 | 0.54 | TcmSP |
| gibberellin 7 | MOL001358 | 73.8 | 0.5 | TcmSP |
| GA77 | MOL001360 | 87.89 | 0.53 | TcmSP |
| GA87 | MOL001361 | 68.85 | 0.57 | TcmSP |
| 3-O-p-coumaroylquinic acid | MOL001368 | 37.63 | 0.29 | TcmSP |
| Amygdalin | MOL001320 | 4.42 | 0.61 | TcmSP |
| luteolin | MOL000006 | 36.16 | 0.25 | TcmSP |
| quercetin | MOL000098 | 46.43 | 0.28 | TcmSP |
| kaempferol | MOL000422 | 41.88 | 0.24 | TcmSP |
| Stigmasterol | MOL000449 | 43.83 | 0.76 | TcmSP |
| CLR | MOL000953 | 37.87 | 0.68 | TcmSP |
| poriferast-5-en-3beta-ol | MOL001771 | 36.91 | 0.75 | TcmSP |
| 4-[(E)-4-(3,5-dimethoxy-4-oxo-1-cyclohexa-2,5-dienylidene)but-2-enylidene]-2,6-dimethoxycyclohexa-2,5-dien-1-one | MOL002694 | 48.47 | 0.36 | TcmSP |
| lignan | MOL002695 | 43.32 | 0.65 | TcmSP |
| Pyrethrin II | MOL002710 | 48.36 | 0.35 | TcmSP |
| 6-Hydroxykaempferol | MOL002712 | 62.13 | 0.27 | TcmSP |
| baicalein | MOL002714 | 33.52 | 0.21 | TcmSP |
| qt_carthamone | MOL002717 | 51.03 | 0.2 | TcmSP |
| quercetagetin | MOL002721 | 45.01 | 0.31 | TcmSP |
| 7,8-dimethyl-1H-pyrimido[5,6-g]quinoxaline-2,4-dione | MOL002757 | 45.75 | 0.19 | TcmSP |
| beta-carotene | MOL002773 | 37.18 | 0.58 | TcmSP |
| Baicalin | MOL002776 | 40.12 | 0.75 | TcmSP |
| hydroxysafflor-yellow-A | MOL002690 | 4.77 | 0.68 | TcmSP |
| ellagic acid | MOL001002 | 43.06 | 0.43 | TcmSP |
| paeoniflorgenone | MOL001918 | 87.59 | 0.37 | TcmSP |
| paeoniflorin | MOL001924 | 53.87 | 0.79 | TcmSP |
| baicalein | MOL002714 | 33.52 | 0.21 | TcmSP |
| Baicalin | MOL002776 | 40.12 | 0.75 | TcmSP |
| beta-sitosterol | MOL000358 | 36.91 | 0.75 | TcmSP |
| sitosterol | MOL000359 | 36.91 | 0.75 | TcmSP |
| Spinasterol | MOL004355 | 42.98 | 0.76 | TcmSP |
| Stigmasterol | MOL000449 | 43.83 | 0.76 | TcmSP |
| (+)-catechin | MOL000492 | 54.83 | 0.24 | TcmSP |
| Ethyl oleate (NF) | MOL002883 | 32.4 | 0.19 | TcmSP |
| campest-5-en-3beta-ol | MOL005043 | 37.58 | 0.71 | TcmSP |
| (2R,3R)-4-methoxyl-distylin | MOL006992 | 59.98 | 0.3 | TcmSP |
| stigmast-7-en-3-ol | MOL006999 | 37.42 | 0.75 | TcmSP |
| FA | MOL000433 | 68.96 | 0.71 | TcmSP |
| Mandenol | MOL001494 | 42 | 0.19 | TcmSP |
| Myricanone | MOL002135 | 40.6 | 0.51 | TcmSP |
| Perlolyrine | MOL002140 | 65.95 | 0.27 | TcmSP |
| wallichilide | MOL002157 | 42.31 | 0.71 | TcmSP |
| poriferasta-7,22E-dien-3beta-ol | MOL001006 | 42.98 | 0.76 | TcmSP |
| 28-norolean-17-en-3-ol | MOL012461 | 35.93 | 0.78 | TcmSP |
| berberine | MOL001454 | 36.86 | 0.78 | TcmSP |
| coptisine | MOL001458 | 30.67 | 0.86 | TcmSP |
| wogonin | MOL000173 | 30.68 | 0.23 | TcmSP |
| delta 7-stigmastenol | MOL002643 | 37.42 | 0.75 | TcmSP |
| beta-daucosterol_qt | MOL000085 | 36.91 | 0.75 | TcmSP |
| quercetin | MOL000098 | 46.43 | 0.28 | TcmSP |
| kaempferol | MOL000422 | 41.88 | 0.24 | TcmSP |
| palmatine | MOL000785 | 64.6 | 0.65 | TcmSP |
| epiberberine | MOL002897 | 43.09 | 0.78 | TcmSP |
| Inophyllum E | MOL003847 | 38.81 | 0.85 | TcmSP |
| beta-sitosterol | MOL000358 | 36.91 | 0.75 | TcmSP |
| Stigmasterol | MOL000449 | 43.83 | 0.76 | TcmSP |
| Catalpol | MOL002819 | 5.07 | 0.44 | TcmSP |
| Gamma-Aminobutyric Acid | MOL000388 | 24.09 | 0.01 | TcmSP |
| Acteoside | MOL003333 | 2.94 | 0.62 | TcmSP |
| luteolin | MOL000006 | 36.16 | 0.25 | TcmSP |
| acacetin | MOL001689 | 34.97 | 0.24 | TcmSP |
| Spinasterol | MOL004355 | 42.98 | 0.76 | TcmSP |
| cis-Dihydroquercetin | MOL004580 | 66.44 | 0.27 | TcmSP |
| Hesperetin | MOL002341 | 70.31 | 0.27 | TcmSP |
| naringenin | MOL004328 | 59.29 | 0.21 | TcmSP |
| nobiletin | MOL005828 | 61.67 | 0.52 | TcmSP |
| Marmin | MOL013381 | 38.23 | 0.31 | TcmSP |
| Linoleyl acetate | MOL001645 | 42.1 | 0.2 | TcmSP |
| Baicalin | MOL002776 | 40.12 | 0.75 | TcmSP |
| isorhamnetin | MOL000354 | 49.6 | 0.31 | TcmSP |
| kaempferol | MOL000422 | 41.88 | 0.24 | TcmSP |
| 3,5,6,7-tetramethoxy-2-(3,4,5-trimethoxyphenyl)chromone | MOL004598 | 31.97 | 0.59 | TcmSP |
| Areapillin | MOL004609 | 48.96 | 0.41 | TcmSP |
| Cubebin | MOL013187 | 57.13 | 0.64 | TcmSP |
| Longikaurin A | MOL004624 | 47.72 | 0.53 | TcmSP |
| (+)-Anomalin | MOL004653 | 46.06 | 0.66 | TcmSP |
| α-spinasterol | MOL004718 | 42.98 | 0.76 | TcmSP |
| petunidin | MOL000490 | 30.05 | 0.31 | TcmSP |
| quercetin | MOL000098 | 46.43 | 0.28 | TcmSP |
| Glycyrol | MOL002311 | 90.78 | 0.67 | TcmSP |
| 7,2',4'-trihydroxy－5-methoxy-3－arylcoumarin | MOL004990 | 83.71 | 0.27 | TcmSP |
| licopyranocoumarin | MOL004904 | 80.36 | 0.65 | TcmSP |
| shinpterocarpin | MOL004891 | 80.3 | 0.73 | TcmSP |
| Phaseol | MOL005017 | 78.77 | 0.58 | TcmSP |
| Licochalcone B | MOL004841 | 76.76 | 0.19 | TcmSP |
| glyasperin F | MOL004810 | 75.84 | 0.54 | TcmSP |
| Inermine | MOL001484 | 75.18 | 0.54 | TcmSP |
| Vestitol | MOL000500 | 74.66 | 0.21 | TcmSP |
| Glyasperins M | MOL005007 | 72.67 | 0.59 | TcmSP |
| (2R)-7-hydroxy-2-(4-hydroxyphenyl)chroman-4-one | MOL004941 | 71.12 | 0.18 | TcmSP |
| 1-Methoxyphaseollidin | MOL004959 | 69.98 | 0.64 | TcmSP |
| formononetin | MOL000392 | 69.67 | 0.21 | TcmSP |
| 3-(3,4-dihydroxyphenyl)-5,7-dihydroxy-8-(3-methylbut-2-enyl)chromone | MOL004863 | 66.37 | 0.41 | TcmSP |
| liquiritin | MOL004903 | 65.69 | 0.74 | TcmSP |
| glyasperin B | MOL004808 | 65.22 | 0.44 | TcmSP |
| Glepidotin B | MOL004829 | 64.46 | 0.34 | TcmSP |
| Licoricone | MOL004855 | 63.58 | 0.47 | TcmSP |
| 1,3-dihydroxy-8,9-dimethoxy-6-benzofurano[3,2-c]chromenone | MOL004914 | 62.9 | 0.53 | TcmSP |
| Glypallichalcone | MOL004835 | 61.6 | 0.19 | TcmSP |
| Glyzaglabrin | MOL004907 | 61.07 | 0.35 | TcmSP |
| Gancaonin G | MOL005000 | 60.44 | 0.39 | TcmSP |
| (2S)-6-(2,4-dihydroxyphenyl)-2-(2-hydroxypropan-2-yl)-4-methoxy-2,3-dihydrofuro[3,2-g]chromen-7-one | MOL004824 | 60.25 | 0.63 | TcmSP |
| 3-(2,4-dihydroxyphenyl)-8-(1,1-dimethylprop-2-enyl)-7-hydroxy-5-methoxy-coumarin | MOL004849 | 59.62 | 0.43 | TcmSP |
| Licoagrocarpin | MOL005003 | 58.81 | 0.58 | TcmSP |
| 8-(6-hydroxy-2-benzofuranyl)-2,2-dimethyl-5-chromenol | MOL004838 | 58.44 | 0.38 | TcmSP |
| Licoagroisoflavone | MOL005012 | 57.28 | 0.49 | TcmSP |
| Mairin | MOL000211 | 55.38 | 0.78 | TcmSP |
| Xambioona | MOL005018 | 54.85 | 0.87 | TcmSP |
| dehydroglyasperins C | MOL005020 | 53.82 | 0.37 | TcmSP |
| 8-prenylated eriodictyol | MOL004993 | 53.79 | 0.4 | TcmSP |
| Glabridin | MOL004908 | 53.25 | 0.47 | TcmSP |
| Glabranin | MOL004910 | 52.9 | 0.31 | TcmSP |
| Glycyrin | MOL004879 | 52.61 | 0.47 | TcmSP |
| Glabrone | MOL004912 | 52.51 | 0.5 | TcmSP |
| licoisoflavanone | MOL004885 | 52.47 | 0.54 | TcmSP |
| Lupiwighteone | MOL003656 | 51.64 | 0.37 | TcmSP |
| Gancaonin A | MOL004856 | 51.08 | 0.4 | TcmSP |
| Jaranol | MOL000239 | 50.83 | 0.29 | TcmSP |
| kanzonols W | MOL004820 | 50.48 | 0.52 | TcmSP |
| Gancaonin H | MOL005001 | 50.1 | 0.78 | TcmSP |
| Odoratin | MOL005016 | 49.95 | 0.3 | TcmSP |
| licochalcone G | MOL004848 | 49.25 | 0.32 | TcmSP |
| Medicarpin | MOL002565 | 49.22 | 0.34 | TcmSP |
| Gancaonin B | MOL004857 | 48.79 | 0.45 | TcmSP |
| Semilicoisoflavone B | MOL004827 | 48.78 | 0.55 | TcmSP |
| 1,3-dihydroxy-9-methoxy-6-benzofurano[3,2-c]chromenone | MOL004913 | 48.14 | 0.43 | TcmSP |
| Calycosin | MOL000417 | 47.75 | 0.24 | TcmSP |
| Quercetin der. | MOL004961 | 46.45 | 0.33 | TcmSP |
| (E)-3-[3,4-dihydroxy-5-(3-methylbut-2-enyl)phenyl]-1-(2,4-dihydroxyphenyl)prop-2-en-1-one | MOL004898 | 46.27 | 0.31 | TcmSP |
| Glabrene | MOL004911 | 46.27 | 0.44 | TcmSP |
| 3'-Methoxyglabridin | MOL004974 | 46.16 | 0.57 | TcmSP |
| Glyasperin C | MOL004811 | 45.56 | 0.4 | TcmSP |
| Isolicoflavonol | MOL004949 | 45.17 | 0.42 | TcmSP |
| Glepidotin A | MOL004828 | 44.72 | 0.35 | TcmSP |
| Isoglycyrol | MOL004948 | 44.7 | 0.84 | TcmSP |
| 2-(3,4-dihydroxyphenyl)-5,7-dihydroxy-6-(3-methylbut-2-enyl)chromone | MOL004866 | 44.15 | 0.41 | TcmSP |
| 3'-Hydroxy-4'-O-Methylglabridin | MOL004966 | 43.71 | 0.57 | TcmSP |
| Eurycarpin A | MOL004915 | 43.28 | 0.37 | TcmSP |
| 7-Methoxy-2-methyl isoflavone | MOL003896 | 42.56 | 0.2 | TcmSP |
| Licoisoflavone | MOL004883 | 41.61 | 0.42 | TcmSP |
| Glycyrrhiza flavonol A | MOL005008 | 41.28 | 0.6 | TcmSP |
| (-)-Medicocarpin | MOL004924 | 40.99 | 0.95 | TcmSP |
| licochalcone a | MOL000497 | 40.79 | 0.29 | TcmSP |
| Inflacoumarin A | MOL004980 | 39.71 | 0.33 | TcmSP |
| (E)-1-(2,4-dihydroxyphenyl)-3-(2,2-dimethylchromen-6-yl)prop-2-en-1-one | MOL004815 | 39.62 | 0.35 | TcmSP |
| 6-prenylated eriodictyol | MOL004989 | 39.22 | 0.41 | TcmSP |
| Licoisoflavone B | MOL004884 | 38.93 | 0.55 | TcmSP |
| 7-Acetoxy-2-methylisoflavone | MOL004991 | 38.92 | 0.26 | TcmSP |
| HMO | MOL004957 | 38.37 | 0.21 | TcmSP |
| sitosterol | MOL000359 | 36.91 | 0.75 | TcmSP |
| (2S)-7-hydroxy-2-(4-hydroxyphenyl)-8-(3-methylbut-2-enyl)chroman-4-one | MOL004945 | 36.57 | 0.32 | TcmSP |
| 2-[(3R)-8,8-dimethyl-3,4-dihydro-2H-pyrano[6,5-f]chromen-3-yl]-5-methoxyphenol | MOL004978 | 36.21 | 0.52 | TcmSP |
| Sigmoidin-B | MOL004935 | 34.88 | 0.41 | TcmSP |
| Licocoumarone | MOL004882 | 33.21 | 0.36 | TcmSP |
| DFV | MOL001792 | 32.76 | 0.18 | TcmSP |
| Kanzonol F | MOL004988 | 32.47 | 0.89 | TcmSP |
| Phaseolinisoflavan | MOL004833 | 32.01 | 0.45 | TcmSP |
| Isotrifoliol | MOL004814 | 31.94 | 0.42 | TcmSP |
| (2S)-2-[4-hydroxy-3-(3-methylbut-2-enyl)phenyl]-8,8-dimethyl-2,3-dihydropyrano[2,3-f]chromen-4-one | MOL004805 | 31.79 | 0.72 | TcmSP |
| icos-5-enoic acid | MOL004985 | 30.7 | 0.2 | TcmSP |
| gadelaidic acid | MOL004996 | 30.7 | 0.2 | TcmSP |
| 5,7-dihydroxy-3-(4-methoxyphenyl)-8-(3-methylbut-2-enyl)chromone | MOL004864 | 30.49 | 0.41 | TcmSP |
| euchrenone | MOL004806 | 30.29 | 0.57 | TcmSP |

**Table S2.** Target proteins of XFZYD

| **Protein name** | **Gene name** | **Prtein name** | **Gene name** |
| --- | --- | --- | --- |
| Progesterone receptor | PGR | Epidermal growth factor | EGF |
| TIF2 | NCOA2 | Retinoblastoma 1 | RB1 |
| Cholinergic receptor, muscarinic 3 | CHRM3 | Tumor necrosis factor | TNF |
| Cholinergic receptor, muscarinic 1 | CHRM1 | CDKN2A | CDKN2A |
| GABA A receptor alpha 2 | GABRA2 | AHSA1 | AHSA1 |
| Gamma-aminobutyric acid receptor, alpha-3 | GABRA3 | ELK 1 | ELK 1 |
| Cholinergic receptor, muscarinic 2 | CHRM2 | I kappa B alpha | NFKBIA |
| Alpha 1B adrenergic receptor | ADRA1B | Cytochrome P-450 reductase | POR |
| GABA-A receptor, alpha 1 | GABRA1 | Ornithine decarboxylase 1 | ODC1 |
| Glutamate receptor, ionotropic, AMPA 2 | GRIA2 | Xanthene dehydrogenase | XDH |
| Gamma aminobutyric acid receptor alpha 6 | GABRA6 | RAF1 | RAF1 |
| Gamma aminobutyric acid receptor alpha 5 | GABRA5 | SOD1 | SOD1 |
| Immunoglobin Gm 1 | IGHG1 | Hypoxia inducible factor 1 alpha subunit | HIF1A |
| Alcohol dehydrogenase 2 | ADH1B | STAT1 | STAT1 |
| Alcohol dehydrogenase 3 | ADH1C | ETO | RUNX1T1 |
| Lysozyme | LYZ | Cyclin E binding protein 1 | HERC5 |
| Prostaglandin G/H synthase 1 | PTGS1 | CDC2 | CDK1 |
| Sodium channel, voltage gated, type 5, alpha subunit | SCN5A | BIP | HSPA5 |
| Cyclooxygenase 2 | PTGS2 | ERBB2 | ERBB2 |
| Retinoid X receptor, alpha | RXRA | Acetyl-CoA carboxylase alpha | ACACA |
| Phosphodiesterase 3A, cGMP inhibited | PDE3A | Cytochrome, subfamily IIIA, polypeptide 4 | CYP3A4 |
| Solute carrier family 6 (neurotransmitter transporter, noradrenalin), member 2 | SLC6A2 | Cytochrome P450 1A2 | CYP1A2 |
| Heat shock protein HSP 90 beta | HSP90AB2P | Caveolin 1 | CAV1 |
| Phosphoinositide 3 kinase, catalytic subunit, gamma | PIK3CG | c-Myc | Myc |
| Potassium channel, voltage gated subfamily H, member 2 | KCNH2 | Coagulation Factor 3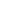 | F3 |
| Protein kinase, cAMP dependent, catalytic, alpha | PRKACA | Gap junction protein, alpha 1, 43kDa | GJA1 |
| Dopamine receptor D1 | DRD1 | Cytochrome p450 subfamily, polypeptide 1 | CYP1A1 |
| Cholinergic receptor, muscarnic 4 | CHRM4 | Interleukin 1, beta | IL1B |
| Serotonin 5-HT-2A receptor | HTR2A | Monocyte chemotactic protein 1 | CCL2 |
| Alpha 1A adrenergic receptor | ADRA1A | Selectin E | SELE |
| Beta-2-adrenergic receptor | ADRB2 | Vascular cell adhesion molecule 1 | VCAM1 |
| Neuronal acetylcholine receptor subunit alpha-2 | CHRNA2 | Prostaglandin E receptor 3, EP3 subtype | PTGER3 |
| Solute carrier family 6 (neurotransmitter transporter, serotonin), member 4 | SLC6A4 | Interleukin 8 | IL8 |
| Mu-type opioid receptor | OPRM1 | Protein kinase C, beta 1 | PRKCB |
| Neuronal acetylcholine receptor subunit alpha-7 | CHRNA7 | Dual oxidase 2 | DUOX2 |
| Bcl 2 | BCL2 | Heat shock protein 27 | HSPB1 |
| Caspase-9 | CASP9 | Estrogen sulfotransferase | SULT1E1 |
| c Jun | JUN | Maltase glucoamylase | MGAM |
| Caspase 3, apoptosis-related cysteine peptidase | CASP3 | Vitamin D (1,25- dihydroxyvitamin D3) receptor | VDR |
| Caspase 8 | CASP8 | Cytochrome P450, subfamily I, polypeptide 1 | CYP1B1 |
| Protein kinase C alpha | PRKCA | Tissue type plasminogen activator | PLAT |
| TGF beta 1 | TGFB1 | Thrombomodulin | THBD |
| Paraoxonase 1 | PON1 | Plasminogen activator inhibitor 1 | SERPINE1 |
| Microtubule associated protein 2 | MAP2 | Collagen, type I, alpha 1 | COL1A1 |
| Mineralocorticoid receptor | NR3C2 | Arachidonate 5 lipoxygenase | ALOX5 |
| Trypsin 1 | PRSS1 | Protein tyrosine phosphatase PTEN | PTEN |
| Carbonic anhydrase II | CA2 | Interleukin 1, alpha | IL1A |
| Coagulation factor X | F10 | Myeloperoxidase | MPO |
| Tyrosine-protein phosphatase non-receptor type 1 | PTPN1 | p47-phox | NCF1 |
| Calmodulin | CALM | ATP-binding cassette sub-family G member 2 | ABCG2 |
| Solute carrier family 6 (neurotransmitter transporter, dopamine), member 3 | SLC6A3 | Hyaluronan synthase 2 | HAS2 |
| Androgen receptor | AR | NFE2L2 | NFE2L2 |
| Dipeptidyl peptidase IV | DPP4 | NAD(P)H dehydrogenase, quinone 1 | NQO1 |
| NFKB3 | RELA | TCDD inducible poly ADP-ribose polymerase | PARP1 |
| EGF receptor | EGFR | Aryl hydrocarbon receptor | AHR |
| AKT1 | AKT1 | Proteasome 26S subunit, non ATPase 3 | PSMD3 |
| VEGF A | VEGFA | Solute carrier family 2 member 4 | SLC2A4 |
| Cyclin D1 | CCND1 | Collagen, type III, alpha 1 | COL3A1 |
| BCL2-like 1 | BCL2L1 | Chemokine, CXC motif, ligand 11 | CXCL11 |
| Cyclin dependent kinase inhibitor 1B | CDKN1B | Macrophage inflammatory protein 2 alpha | CXCL2 |
| Matrix metalloprotease 2 | MMP2 | WD repeat domain 22 | DCAF5 |
| Matrix metalloproteinase 9 | MMP9 | NR1I3 | NR1I3 |
| ERK2 | MAPK1 | CHK2 checkpoint homolog | CHEK2 |
| Cell division cycle associated 1 | NUF2 | Claudin 4 | CLDN4 |
| Matrix metalloproteinase 1 | MMP1 | Peroxisome proliferator activated receptor, alpha | PPARA |
| Interleukin 6 | IL6 | Peroxisome proliferator activated receptor, delta | PPARD |
| Interleukin-4 | IL4 | Heat-shock transcription factor 1 | HSF1 |
| Interleukin-2 | IL2 | C reactive protein | CRP |
| Interleukin-10 | IL10 | Interferon-gamma-inducible protein 10 | CXCL10 |
| Interferon, gamma | IFNG | IKK alpha | CHUK |
| Intercellular adhesion molecule 1 | ICAM1 | Osteopontin | SPP1 |
| Insulin receptor | INSR | Runt related transcription factor 2 | RUNX2 |
| Myeloid cell leukemia 1 | MCL1 | Ras association domain family protein 1 | RASSF1 |
| Hepatocyte growth factor receptor | MET | E2F transcription factor 1 | E2F1 |
| Heme oxygenase 1 | HMOX1 | E2F Transcription factor 2 | E2F2 |
| Glutathione S transferase 3 | [GSTP1](http://www.genenames.org/data/hgnc_data.php?hgnc_id=4638) | Prostatic acid phosphatase | ACPP |
| Cyclin B1 | CCNB1 | Cathepsin D | CTSD |
| MDM2 | MDM2 | IGF binding protein 3 | IGFBP3 |
| DNA topoisomerase II alpha | TOP2A | Insulin like growth factor II | IGF2 |
| DNA topoisomerase I | TOP1 | Interferon regulatory factor 1 | IRF1 |
| TP53 | p53 | ErbB3 | ERBB3 |
| Cyclin dependent kinase 4 | CDK4 | Deiodinase, iodothyronine, type I | DIO1 |
| Tumor necrosis factor ligand superfamily member 5 | CD40LG | Procollagen C endopeptidase enhancer | PCOLCE |
| Caspase 7 | CASP7 | Aminopeptidase puromycin sensitive | NPEPPS |
| Survivin | BIRC5 | Hexokinase 2 | HK2 |
| Apoptosis inhibitor 3 | XIAP | NKX3.1 | NKX3-1 |
| Amyloid beta A4 protein | APP | Synaptic Ras GTPase activating protein 1 | SYNGAP1 |
| Adenylate cyclase 2 | ADCY2 | Glutathione S-transferase Mu-1 | GSTM1 |
| Peroxisome proliferator-activated receptor gamma | PPARG | Glutathione S-transferase Mu-2 | GSTM2 |
| Aldose reductase | AKR1B1 | Nitric oxide synthase 2A | NOS2 |
| Prothrombin | F2 | IKK beta | IKBKB |
| Matrix metalloproteinase 3 | MMP 3 | Mitogen-activated protein kinase 8 | MAPK8 |
| Coagulation factor VII | F7 | Calcineurin A alpha | PPP3CA |
| Nitric oxide synthase 3 | NOS3 | Aldo keto reductase family 1, member C3 | AKR1C3 |
| Acetylcholinesterase | ACHE | Secretory leukocyte protease inhibitor | SLPI |
| Monoamine oxidase B | MAOB | Steroid receptor coactivator 1 | NCOA1 |
| c-Fos | FOS | Alpha 2A adrenergic receptor | ADRA2A |
| Eukaryotic translation initiation factor 6 | EIF6 | Leukotriene A4 hydrolase | LTA4H |
| BAX | BAX | Monoamine oxidase A | MAOA |
| Plasminogen activator urokinase | PLAU | Chymotrypsinogen B1 | CTRB1 |
| Adrenergic receptor, Beta 1 | ADRB1 | Trypsinogen IV | PRSS3 |
| Estrogen receptor alpha | ESR1 | Gamma Aminobutyrate Transaminase | ABAT |
| Potassium large conductance calcium activated channel subfamily M alpha member 1 | KCNMA1 | Alcohol dehydrogenase 1 | ADH1A |
| Fos-like antigen 1 | FOSL1 | Valosin containing protein | VCP |
| FRA2 | FOSL2 | Solute carrier family 6 (neurotransmitter transporter, GABA), member 1 | SLC6A1 |
| Cytochrome C | CYCS | Fatty acid synthase | FASN |
| Arachidonate 12-oxidoreductase | ALOX12 | FAS ligand | FASLG |
| Nuclear factor of activated T-cells, cytoplasmic, calcineurin-dependent 1 | NFATC1 | Cytochrome P450, subfamily XIX | CYP19A1 |
| Tudor repeat associator with PCTAIRE 2 | TDRD7 | ERK1 | MAPK3 |
| HIF prolyl hydroxylase 2 | EGLN1 | Low density lipoprotein receptor | LDLR |
| NADPH oxidase, EF-hand calcium binding domain 5 | NOX5 | BCL2 antagonist of cell death | BAD |
| Fatty acid binding protein 5 | FABP5 | Microsomal triglyceride transfer protein, large subunit | MTTP |
| Apolipoprotein D | APOD | Apolipoprotein B | APOB |
| Albumin | ALB | Phospholipase B1 | PLB1 |
| Catenin beta | CTNNB1 | HMG CoA reductase | HMGCR |
| Matrix metalloproteinase 10 | MMP10 | UDP glycosyltransferase 1 family, polypeptide A1 | UGT1A1 |
| NFKB1 | NFKB1 | Sterol regulatory element binding transcription factor 1 | SREBF1 |
| Cyclin dependent kinase 2 | CDK2 | Glutathione reductase | GSR |
| Glutathione S-transferase alpha 1 | GSTA1 | ATP binding cassette subfamily C member1 | ABCC1 |
| Glutathione S-transferase, alpha-2 | GSTA2 | ACRP30 | ADIPOQ |
| CD14 | CD14 | Sterol O acyltransferase 2 | SOAT2 |
| Lipopolysaccharide binding protein | LBP | Aldo-keto reductase family 1 member C1 | AKR1C1 |
| Mitochondrial ribosomal protein L56 | LACTB | Carboxylesterase 1 | CES1 |
| Catalase | CAT | Sterol O-Acyltransferase | SOAT1 |
| Glycogen synthase kinase 3 beta | GSK3B | Tissue inhibitor of metalloproteinase 1 | TIMP1 |
| VEGF receptor 2 | KDR | Active transcription factor CREB | CREB1 |
| Estrogen receptor beta | ESR2 | Cytosolic phospholipase A2 | PLA2G4A |
| MAPK14 | MAPK14 | CD163 | CD163 |
| Cell cycle checkpoint kinase | CHEK1 | EphB2 | EPHB2 |
| PIM1 | PIM1 | Glycogen phosphorylase | PYGM |
| Cyclin A2 | CCNA2 | Oxidized low density lipoprotein receptor 1 | OLR1 |
| Glucocorticoid receptor | NR3C1 | 5-hydroxytryptamine (serotonin) receptor 3A | HTR3A |
| Phosphodiesterase 10A | PDE10A | Opioid receptor | OPRD1 |
| BCL2 binding component 3 | BBC3 | Alpha 1D adrenergic receptor | ADRA1D |
| Telomerase protein component 1 | TEP1 | Retinoid X receptor, beta | RXRB |
| Protein kinase C delta | PRKCD | Protein kinase inhibitor alpha | PKIA |
| Fibronectin 1 | FN1 | Sirtuin 1 | SIRT1 |
| Butyrylcholinesterase | BCHE | ATP synthase, beta | ATP5B |
| Glutamate oxaloacetate transaminase-1 | GOT1 | NADH dehydrogenase 6 | MT-ND6 |
| AGAT | GATM | 3-beta hydroxysteroid dehydrogenase, delta isomerase, type 2 | HSD3B2 |
| 3-beta-HSD, placental type | HSD3B1 | STAT3 | STAT3 |
| Cholinergic receptor, muscarnic 5 | CHRM5 | Beta site App cleaving enzyme | BACE1 |
| Mitogen-activated protein kinase 10 | MAPK10 |  |  |

**Table S3.** TBI specific proteins.

| **Protein name** | **Gene name** | **Protein name** | **Gene name** |
| --- | --- | --- | --- |
| Dopamine receptor D2 | DRD2 | Calcium channel, voltage dependent, beta 3 subunit | CACNB3 |
| Syntaxin 1A | STX1A | Protein phosphatase 1A magnesium dependent alpha isoform | PPM1A |
| Synuclein alpha | SNCA | Calcium channel, voltage dependent, N type, alpha 1B subunit | CACNA1B |
| HIC5 | HIC5 | Angiotensin I converting enzyme | ACE |
| Protein kinase C, alpha binding protein | AKT1 | Angiotensin receptor 1 | AGTR1 |
| Solute carrier family 6 (neurotransmitter transporter, dopamine), member 3 | SLC6A3 | G protein dependent receptor kinase 2 | ADRBK1 |
| RACK1 | RACK1 | Beta adrenergic receptor kinase 2 | ADRBK2 |
| Actinin alpha 1 | ACTN1 | G protein coupled receptor kinase 4 | GRK4 |
| Adaptor related protein complex 4, mu 1 subunit | AP4M1 | Guanine nucleotide binding protein, alpha 11 | GNA11 |
| AP47 | AP47 | Nitric oxide synthase 3 | NOS3 |
| CASK | CASK | Nitric oxide synthase 1 | NOS1 |
| DAP1 | DAP1 | Protein kinase C delta | PRKCD |
| Fyn | Fyn | G protein coupled receptor kinase 6 | GRK6 |
| Glutamate receptor, ionotropic, N-methyl D-aspartate 1 | GRIN1 | G protein coupled receptor kinase 5 | GRK5 |
| Phospholipase C, gamma 1 | PLCG1 | Albumin | ALB |
| Regulator of G protein signaling 3 | RGS3 | Amyloid beta A4 protein | APP |
| Cyclin dependent kinase 5 | CDK5 | Ciliary neurotrophic factor | CNTF |
| N-methyl D-aspartate receptor subunit 2A | GRIN2A | Macroglobulin, alpha 2 | A2M |
| Protein tyrosine phosphatase, non receptor type, 4 | PTPN4 | Microtubule associated protein tau | MAPT |
| Protein kinase C alpha | PRKCA | Apolipoprotein E | APOE |
| c-Src | SRC | Low density lipoprotein receptor-related protein 1 | LRP1 |
| Synapse associated protein 102 | DLG3 | Cathepsin B | CTSB |
| PTK2B protein tyrosine kinase 2 beta | PTK2B | Neurofilament 3 | NEFM |
| Interleukin 16 | IL16 | Phospholipid transfer protein | PLTP |
| PATJ | PATJ | Prion protein | PRNP |
| Channel associated protein of synapse 110 | [DLG2](http://www.genenames.org/data/hgnc_data.php?hgnc_id=2901) | VLDL receptor | VLDLR |
| CaMK II alpha subunit | CAMK2A | Megalin | LRP2 |
| Glutamate receptor, ionotropic, N-methyl-D-aspartate 3A | GRIN3A | Scavenger receptor class B member 1 | SCARB1 |
| Glutamate receptor, ionotropic, N-methyl-D-aspartate 3B | GRIN3B | Low density lipoprotein receptor | LDLR |
| CaM kinase II beta subunit | CAMK2B | Alpha 1B adrenergic receptor | ADRA1B |
| RICS | RICS | Apolipoprotein A I | APOA1 |
| Synapse associated protein 90 | DLG4 | Senataxin | SETX |
| Synapse associated protein 97 | DLG1 | Potassium large conductance calcium activated channel subfamily M alpha member 1 | KCNMA1 |
| Calmodulin | CALM | cAMP-specific 3`,5`-cyclic phosphodiesterase 4B | PDE4B |
| ErbB4 | ERBB4 | Cathepsin L | CTSL1 |
| A-kinase anchor protein 9 | AKAP9 | CDC45 | CDC45 |
| Actinin alpha 2 | ACTN2 | Connexin 47 | GJC2 |
| Cadherin 2 | CDH2 | CUB domain containing protein 1 | CDCP1 |
| Calmodulin 1 | CALM1 | Damage-specific DNA binding protein 1, 127kDa | DDB1 |
| Calnexin | CANX | Fibrinogen, alpha chain | FGA |
| Catenin beta | CTNNB1 | Fibronectin 1 | FN1 |
| Citron | CIT | GABA-B receptor | GABBR1 |
| Clathrin, heavy polypeptide | CLTC | Haptoglobin | HP |
| Dopamine receptor D1 | DRD1 | Immunoglobin Gm 1 | IGHG1 |
| Dual specificity phosphatase 4 | DUSP4 | Keratin 10 | KRT10 |
| Dynamin 1 | DNM1 | Keratin 14 | KRT14 |
| EphB2 | EPHB2 | Keratin 9 | KRT9 |
| EphB4 | EPHB4 | Keratin 1 | KRT1 |
| FUS | FUS | Olfactory receptor, family 8, subfamily D, member 2 | OR8D2 |
| H-Ras | HRAS | Kallikrein 3, (prostate specific antigen) | KLK3 |
| Heat shock 70 KD protein 1A | HSPA1A | ATM | ATM |
| Heterogeneous nuclear ribonucleoprotein U | HNRNPU | Olfactory receptor family 2 subfamily T member 6 | OR2T6 |
| Internexin, alpha | INA | Talin 2 | TLN2 |
| MAP2K2 | MAP2K2 | Transthyretin | TTR |
| Microtubule associated protein 2 | MAP2 | Calcium channel, voltage-dependent, alpha 1I subunit | CACNA1I |
| Myosin heavy chain 9, nonmuscle | MYH9 | Zinc finger protein 232 | ZNF232 |
| N-methyl D-aspartate receptor subunit 2B | GRIN2B | PLAG1 | PLAG1 |
| Neurofibromatosis type I | NF1 | Rlf protein involved in activation of LMYC | RLF |
| Neurofilament light polypeptide | NEFL | Alpha-2-HS glycoprotein | AHSG |
| NMDAR2D | GRIN2D | Apolipoprotein A IV | APOA4 |
| Protein kinase C epsilon | PRKCE | Apolipoprotein C III | APOC3 |
| Protein kinase C, beta 1 | PRKCB | Crumbs protein homolog 1 | CRB1 |
| Protein kinase C, gamma | PRKCG | SEC15 like 1 | EXOC6 |
| Protein phosphatase 2 regulatory subunit B alpha | PPP2R2A | Serine arginine rich pre-mRNA splicing factor SR-A1 | SCAF1 |
| Ras related protein 2A | RAP2A | Glial fibrillary acidic protein | GFAP |
| Ribosomal protein S6 kinase alpha 3 | RPS6KA3 | TSC22 domain family, member 1 | TSC22D1 |
| Spectrin, alpha, non-erythrocytic 1 (alpha-fodrin) | SPTAN1 | Immunoglobulin kappa light chain | IGKC |
| Tight junction protein 1 | TJP1 | Integrin, beta 5 | ITGB5 |
| TRAF3 | TRAF3 | Keratin 6A | KRT6A |
| Transcription factor Sp3 | SP3 | Plasma protease C1 inhibitor | SERPING1 |
| Synaptic Ras GTPase activating protein 1 | SYNGAP1 | C20ORF121 protein | TTPAL |
| Serine/threonine protein phosphatase 2A, 65 kDa regulatory subunit A, alpha isoform | PPP2R1A | Prothrombin | F2 |
| Nanos homolog 1 | NANOS1 | AMP deaminase 3 | AMPD3 |
| Discs large associated protein 4 | DLGAP4 | Apolipoprotein A II | APOA2 |
| Tubulin, alpha 4 | TUBA4 | Apolipoprotein C-I | APOC1 |
| Tissue type plasminogen activator | PLAT | Apolipoprotein C II | APOC2 |
| Low density lipoprotein receptor-related protein 8, apolipoprotein e receptor | LRP8 | Choline kinase beta | CHKB |
| Protein phosphatase 2A, catalytic subunit, beta isoform | PPP2CB | Coagulation factor VII | F7 |
| Calpain, large polypeptide L1 | CAPN1 | Complement factor B | CFB |
| CaM kinase II gamma subunit | CAMK2G | Complement factor D | CFD |
| CaMK II delta subunit | CAMK2D | Complement factor H | CFH |
| Clathrin adaptor complex AP2, MU subunit | AP2M1 | Cystatin C | CST3 |
| SHP2 | [PTPN11](http://www.genenames.org/data/hgnc_data.php?hgnc_id=9644) | Dermcidin | DCD |
| Phosphatidylinositol 3 kinase, catalytic subunit alpha | PIK3CA | Gelsolin | GSN |
| Parkinson disease (autosomal recessive, juvenile) 2, parkin | PARK2 | NOPE | NOPE |
| Veli 1 | LIN7A | Hemopexin | HPX |
| Veli 2 | LIN7B | Polyhomeotic like 3 | PHC3 |
| MAGI-3 | MAGI3 | Hsc70 interacting protein | ST13 |
| Exocyst complex component 4 | EXOC4 | Der1 like domain family, member 1 | DERL1 |
| Calcium/calmodulin dependent protein kinase II inhibitor 1 | CAMK2N1 | C19orf16 protein | FAM71E2 |
| TANC | TANC | Queuine tRNA ribosyltransferase domain containing 1 | QTRTD1 |
| APC | APC | KIAA0232 gene product | KIAA0232 |
| Densin180 | LRRC7 | Inter alpha trypsin inhibitor heavy chain H1 | ITIH1 |
| ABL | ABL | Keratin 13 | KRT13 |
| Guanine nucleotide binding protein, alpha `other` | GNAO1 | Keratin 16 | KRT16 |
| Guanine nucleotide binding protein, beta 1 | GNB1 | Keratin 5 | KRT5 |
| RIMS1 | RIMS1 | Keratin 6B | KRT6B |
| MINT1 | MINT1 | Obscurin-like 1 | OBSL1 |
| Regulator of G protein signaling 12 | RGS12 | MGEA6 | MGEA6 |
| Enigma like lim domain protein | PDLIM5 | Aspartylglucosaminidase | AGA |
| NCOA3 | NCOA3 | Alpha 1 antichymotrypsin | SERPINA3 |
| Olfactory receptor family 3 subfamily A member 2 | OR3A2 | Calreticulin | CALR |
| Beta thromboglobulin | PPBP | Cathepsin D | CTSD |
| Platelet factor 4, variant 1 | PF4V1 | Cholinergic receptor neuronal nicotinic alpha polypeptide 7 | CHRNA7 |
| Protocadherin 1 | PCDH1 | Collagen, type IV, alpha 3 | COL4A3 |
| Ran binding protein 2 | RANBP2 | Collagen, type IV, alpha 2 | COL4A2 |
| Ryanodine receptor 2 | RYR2 | Collagen, type IV, alpha 1 | COL4A1 |
| Sacsin | SACS | Collagen, type I, alpha 2 | COL1A2 |
| SH3 domain binding protein 5 | SH3BP5 | Nidogen 1 | NID1 |
| Phospholipase A2, group IVF | PLA2G4F | Fibulin 1 | FBLN1 |
| T cell lymphoma invasion and metastasis 1 | TIAM1 | Glyceraldehyde 3 phosphate dehydrogenase | GAPDH |
| Trypsinogen IV | PRSS3 | Heme oxygenase 2 | HMOX2 |
| Zinc finger protein 292 | ZNF292 | Perlecan | HSPG2 |
| Adapter related protein complex 4 epsilon 1 subunit | AP4E1 | Hydroxyacyl dehydrogenase, subunit B | HADHB |
| Cdk5 and Abl enzyme substrate 1 | CABLES1 | Insulin degrading enzyme | IDE |
| Solute carrier family 25, member 13 | SLC25A13 | Laminin, alpha 1 | LAMA1 |
| CARD12 | CARD12 | Kininogen | KNG1 |
| Calcium activated chloride channel 2 | CLCA2 | Coagulation factor XII | F12 |
| Diacylglycerol kinase, gamma, 90 kd | DGKG | BAP31 | BAP31 |
| Dystrophin | DMD | Biglycan | BGN |
| Dicer1, Dcr-1 homolog | DICER1 | Collagen type IV, alpha 5 | COL4A5 |
| GRID | GRID | Collagen type IV, alpha 6 | COL4A6 |
| FLJ12716 protein | C4orf41 | Kinesin 2 | KLC1 |
| C9orf36 protein | FAM75A7 | Caspase 3, apoptosis-related cysteine peptidase | CASP3 |
| Family with sequence similarity 113, member B | FAM113B | Caspase 6 | CASP6 |
| PDZ domain containing RING finger 4 | PDZRN4 | Trypsinogen 2 | PRSS2 |
| PALB2 | PALB2 | Caspase 8 | CASP8 |
| Chromosome 12 open reading frame 35 | C12orf35 | Bleomycin hydrolase | BLMH |
| LIM domain binding 3 | LDB3 | MINT1 | MINT1 |
| KIAA1712 | KIAA1712 | Synuclein beta | SNCB |
| Integrin alpha 2 | ITGA2 | Caspase 4 | CASP4 |
| Jumonji | JARID2 | Amyloid beta A4 precursor protein binding, family B, member 1 | APBB1 |
| GCN1 general control of amino acid synthesis 1 like 1 | GCN1L1 | APBB2 | APBB2 |
| Calmodulin binding transcription activator 1 | CAMTA1 | Amyloid beta (A4) precursor protein binding family B member 3 | APBB3 |
| Solute carrier family 9 (sodium/hydrogen exchanger) isoform 8 | SLC9A8 | APBA2 | APBA2 |
| CCR4NOT transcription complex subunit 1 | CNOT1 | Amyloid beta precursor protein binding protein 1 | NAE1 |
| Linker for activation of T cells | LAT | Disabled-1 | DAB1 |
| Myosin, light polypeptide 4, alkali; atrial, embryonic | MYL4 | Numb homolog | NUMB |
| Myosin light chain kinase | MYLK3 | APBA3 | APBA3 |
| Titin | TTN | MAPK8 interacting protein 1 | MAPK8IP1 |
| Nephrin | NPHS1 | Glycogen synthase kinase 3 beta | GSK3B |
| Sodium dependent neutral amino acid transporter type 2 | SLC1A5 | Nicastrin | NCSTN |
| PEG3 | PEG3 | PAT1 | PAT1 |
| Carnitine O-octanoyltransferase | CROT | Beta site APP cleaving enzyme 2 | BACE2 |
| BCL2 binding component 3 | BBC3 | Humanin | MT-RNR2 |
| Ran binding protein 3 | RANBP3 | Collagen type XXV alpha 1 | COL25A1 |
| Sodium channel, voltage gated, type 5, alpha subunit | SCN5A | Tumor suppressor p53 binding protein 2 | TP53BP2 |
| Spastin | SPAST | Spondin 1 extracellular matrix protein | SPON1 |
| Src like adapter protein 2 | SLA2 | Low density lipoprotein related protein 1B | LRP1B |
| TRAP150 | TRAP150 | Beta amyloid binding protein | TM2D1 |
| Shugoshin like 2 | SGOL2 | Erythrocyte membrane protein band 4.1 | EPB41 |
| Vinexin beta | SORBS3 | Clusterin | CLU |
| ABL | ABL | HtrA serine peptidase 2 | HTRA2 |
| Adaptor protein containing PH and SH2 domains | SH2B2 | HIV-1 Tat interacting protein, 60kDa | KAT5 |
| ERK1 | MAPK3 | Hydroxyacyl CoA dehydrogenase, type II | HSD17B10 |
| Grb2 | GRB2 | Glypican 1 | GPC1 |
| Nerve growth factor receptor | NGFR | Integral membrane protein 2B | ITM2B |
| Phosphatidylinositol 3 kinase regulatory subunit, alpha | PIK3R1 | Beta site App cleaving enzyme | BACE1 |
| Protein tyrosine phosphatase, non-receptor type 1 | PTPN1 | LDL receptor adaptor protein | LDLRAP1 |
| Ras related protein 1A | RAP1A | X11L binding protein 51 | NECAB3 |
| RasGAP | RASA1 | TGF beta 2 | TGFB2 |
| Sequestome 1 | SQSTM1 | Pin1 | PIN1 |
| SHC (Src homology 2 domain containing) transforming protein 1 | SHC1 | Peptidyl-prolyl isomerase D | PPID |
| TID 1 | DNAJA3 | APBA2 | APBA2 |
| Ubiquitin protein ligase NEDD4 like | NEDD4L | SMAD4 | SMAD4 |
| Tyrosine kinase receptor A | NTRK1 | SMAD3 | SMAD3 |
| Csk homologous kinase | MATK | CCCTC binding factor | CTCF |
| Caveolin 1 | CAV1 | Syntaxin binding protein 1 | STXBP1 |
| Dynein light chain 1 | DYNLL1 | Heat shock 70 kDa protein 8 | HSPA8 |
| GIPC PDZ domain containing family, member 1 | GIPC1 | HSP90A | HSP90A |
| FRS2 | FRS2 | N ethylmaleimide sensitive factor | NSF |
| FRS3 | FRS3 | Cyclophilin A | PPIA |
| Nerve growth factor, beta | NGF | Tubulin, beta | TUBB |
| RUSC1 | RUSC1 | Actin beta | ACTB |
| SHC transforming protein 3 | SHC3 | Myelin basic protein | MBP |
| RICS | RICS | 14-3-3 zeta | YWHAZ |
| SH2 B homolog | SH2B1 | Ubiquitin carboxyl terminal esterase L1 | UCHL1 |
| KIDINS220 | KIDINS220 | Phosphoglycerate mutase 1 | PGAM1 |
| CRK | CRK | Uracil DNA glycosylase | UNG |
| IRS2 | IRS2 | ASK1 | ASK1 |
| IRS 1 | IRS1 | Caspase 1 | CASP1 |
| Ubiquitin B | UBB | Phosphorylase kinase muscle gamma 1 | PHKG1 |
| SHC (Src homology 2 domain containing) transforming protein 2 | SHC2 | S100 calcium binding protein, beta | S100B |
| Ras protein specific guanine nucleotide releasing factor 1 | RASGRF1 | Spectrin, beta I | SPTB |
| PLC, gamma 2 | PLCG2 | Tubulin alpha 1 | TUBA4A |
| SHC | SHC | O-linked N-acetylglucosamine transferase | OGT |
| Guanine nucleotide releasing factor 2 | RAPGEF1 | Protein phosphatase 5 catalytic subunit | PPP5C |
| CRKL | CRKL | Serine threonine protein kinase N | PKN1 |
| GAB2 | GAB2 | 14-3-3 Beta | YWHAB |
| TRAF6 | TRAF6 | Protein phosphatase 2, regulatory subunit B (B56), alpha | PPP2R5A |
| Acetylcholinesterase | ACHE | Staufen | STAU1 |
| Crystallin, alpha B | CRYAB | Caspase 7 | CASP7 |
| Flotillin 1 | FLOT1 | MAPK12 | MAPK12 |
| Homer 3 | HOMER3 | Glycogen synthase kinase 3 alpha | GSK3A |
| Homer homolog 2 | HOMER2 | MARK | MARK |
| JNK interacting protein 2 | MAPK8IP2 | Protein phosphatase 2A, catalytic subunit, alpha isoform | PPP2CA |
| NUMBL | NUMBL | MAP/microtubule affinity regulating kinase 4 | MARK4 |
| Presenilin 1 | [PSEN1](http://www.genenames.org/data/hgnc_data.php?hgnc_id=9508) | Apoptosis antagonizing transcription factor | AATF |
| Presenilin 2 | PSEN2 | CDC2 | CDK1 |
| TGF beta 1 | TGFB1 | Hypothetical protein FLJ10357 | LOC55701 |
| STIP1 homologous and U box containing protein 1 | STUB1 | Ku antigen, 70kDa | XRCC6 |
| Ribosomal protein S6 kinase, 70kDa, polypeptide 1 | RPS6KB1 | Profilin 2 | PFN2 |
| ERK2 | MAPK1 | Transcription factor Sp1 | SP1 |
| Neurofilament protein, heavy polypeptide | NEFH | Transglutaminase 2 | TGM2 |
| Ubiquitin conjugating enzyme E2D2 | UBE2D2 | p53 | p53 |
| Colony stimulating factor 1 | CSF1 | Cystathionine beta synthase | CBS |
| Decorin | DCN | Factor VIII associated gene 1 | F8A1 |
| Proteinase 3 | PRTN3 | TATA box binding protein | TBP |
| Tumor necrosis factor receptor 1 | TNFRSF1A | MAP3K10 | MAP3K10 |
| Tumor necrosis factor receptor 2 | TNFRSF1B | CREBBP | CREBBP |
| TRAF2 | TRAF2 | Nuclear receptor corepressor 1 | NCOR1 |
| Matrix metalloproteinase 17 | MMP17 | Huntingtin associated protein 1 | HAP1 |
| ADAM metallopeptidase domain 9 | ADAM9 | HIP1 | HIP1 |
| ADAM 17 | ADAM17 | Optineurin | OPTN |
| TNF alpha | TNF | Collapsin response mediator protein 1 | CRMP1 |
| Interferon, gamma | IFNG | C-terminal binding protein 1 | CTBP1 |
| TNF beta | LTA | Ubiquitin conjugating enzyme E2-25K | UBE2K |
| Lymphotoxin beta TNF superfamily member 3 | LTB | SH3 containing GRB2 like protein 3 | SH3GL3 |
| Lymphotoxin B receptor | LTBR | Sin3 associated polypeptide ,30KD | SAP30 |
| Tumor necrosis factor receptor superfamily, member 14 | TNFRSF14 | IKAP | IKAP |
| Galectin 2 | LGALS2 | Protein kinase C and casein kinase substrate in neurons 1 | PACSIN1 |
| Uromodulin | UMOD | Adaptor related protein complex 2, alpha2 subunit | AP2A2 |
| PDLIM1 interacting kinase 1 like | PDIK1L | GIT1 | GIT1 |
| Serum/glucocorticoid regulated kinase | SGK1 | GASP2 | GASP2 |
| Fanconi anemia, complementation group D2 | FANCD2 | UTP14, U3 small nucleolar ribonucleoprotein, homolog A | UTP14A |
| Keratin 18 | KRT18 | PIASY | PIASY |
| Casein kinase 1, alpha 1 | CSNK1A1 | RE1 silencing transcription factor | REST |
| STAT1 | STAT1 | Chromodomain helicase DNA binding protein 3 | CHD3 |
| TRAF and TNF receptor associated protein | TTRAP | Symplekin | SYMPK |
| TRAF interacting protein | TRAIP | Fasciculation and elongation protein zeta 1 (zygin I) | FEZ1 |
| Bone marrow kinase BMX | BMX | SIN3A | SIN3A |
| TRAF1 | TRAF1 | HIP14 | HIP14 |
| Smad ubiquitination regulatory factor 2 | SMURF2 | Transcription elongation regulator 1 | TCERG1 |
| HSPC142 protein | C19orf62 | Huntingtin interacting protein 12 | HIP1R |
| Growth factor independent 1B | GFI1B | Formin binding protein 3 | PRPF40A |
| 14-3-3 gamma | YWHAG | Huntingtin interacting protein C | PRPF40B |
| 14-3-3 theta | YWHAQ | TBP associated factor 4 | TAF4 |
| Calcineurin A alpha | PPP3CA | Metastasis suppressor 1 | MTSS1 |
| Lysyl tRNA synthetase | KARS | Huntingtin interacting protein E | FICD |
| SOD1 | SOD1 | Huntingtin interacting protein K | C15orf63 |
| Bcl 2 | BCL2 | Mediator of RNA polymerase II transcription, subunit 31 homolog | MED31 |
| Copper chaperone for superoxide dismutase | [CCS](http://www.genenames.org/data/hgnc_data.php?hgnc_id=1613) | SH3 domain GRB2 like endophilin B1 | SH3GLB1 |
| Heat shock 40 kDa protein 1 | DNAJB1 | KIAA1377 protein | KIAA1377 |
| RING finger protein 19 | RNF19A | Huntingtin interacting protein M | CXorf27 |
| HOX B2 | HOXB2 | Dynactin 1 | DCTN1 |
| EGF receptor | EGFR | A kinase PRKA anchor protein 8 like | AKAP8L |
| Pyruvate dehydrogenase kinase, isoenzyme 2 | PDK2 | N-methyl D-aspartate receptor subunit 2A | GRIN2A |
| SMT3 suppressor of mif two 3 homolog 1 (yeast) | SUMO1 | Glutamate receptor, ionotropic, N-methyl D-aspartate 1 | GRIN1 |
| Translocated promoter region | TPR | N-methyl D-aspartate receptor subunit 2B | GRIN2B |
| TRIP10 | TRIP10 | NMDAR2D | GRIN2D |
| Calcium channel, voltage dependent, alpha 2/delta subunit 2 | CACNA2D2 | N-methyl D-aspartate receptor subunit 2C | GRIN2C |
| Calcium channel, voltage dependent, alpha 2/delta subunit 3 | CACNA2D3 | Solute carrier family 1, member 3 | SLC1A3 |
| B2 bradykinin receptor | BDKRB2 | Uncoupling protein 2 | UCP2 |
| Huntingtin | HTT |  |  |

**Table S4.** 119 potential compounds of XFZYD for treating TBI

| **Molecule**  **Number** | **PubChem CID** | **Compound** | **OB** | **DL** | **Degree** | **Betweenness** | **Structure** |
| --- | --- | --- | --- | --- | --- | --- | --- |
| Mol 148 | 5280343 | Quercetin | 46.43 | 0.28 | 153 | 0.359 | 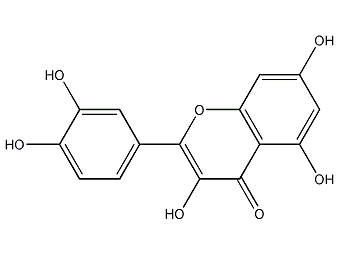 |
| Mol 108 | 5280863 | Kaempferol | 41.88 | 0.24 | 65 | 0.077 | 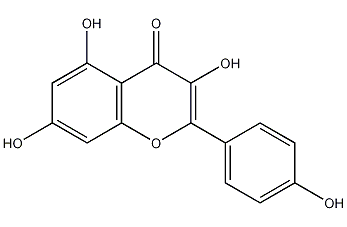 |
| Mol 127 | 5280445 | Luteolin | 36.16 | 0.25 | 48 | 0.057 | 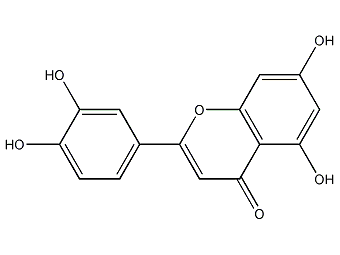 |
| Mol 160 | 5281703 | Wogonin | 30.68 | 0.23 | 46 | 0.04 | 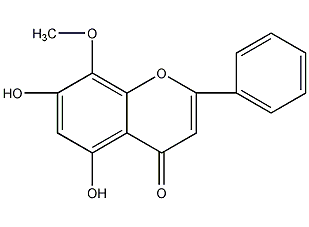 |
| Mol 33 | 5326329 | 7-Methoxy-2-methyl isoflavone | 42.56 | 0.2 | 44 | 0.019 | 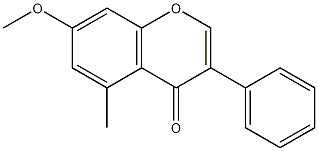 |
| Mol 45 | 222284 | Beta-sitosterol | 36.91 | 0.75 | 41 | 0.041 | 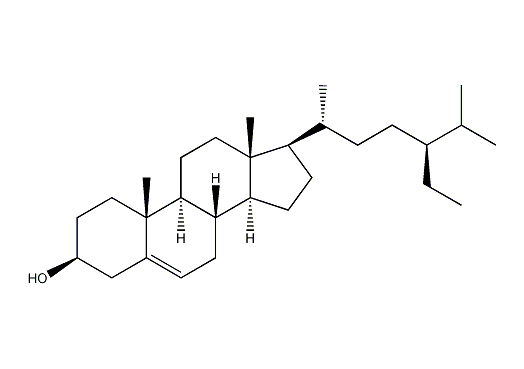 |
| Mol 40 | 5281605 | Baicalein | 33.52 | 0.21 | 40 | 0.055 | 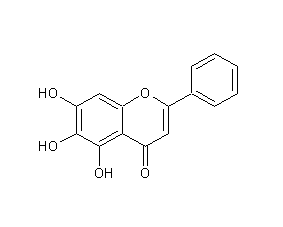 |
| Mol 63 | 5280378 | Formononetin | 69.67 | 0.21 | 39 | 0.031 | 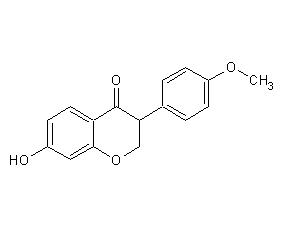 |
| Mol 133 | 932 | Naringenin | 59.29 | 0.21 | 39 | 0.088 | 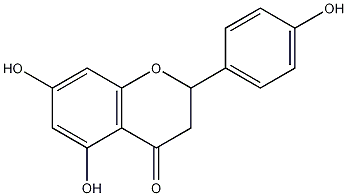 |
| Mol 105 | 5281654 | Isorhamnetin | 49.6 | 0.31 | 38 | 0.022 | 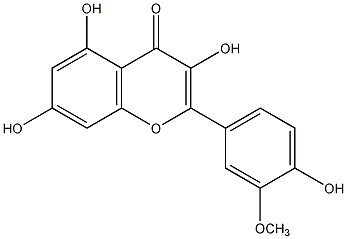 |
| Mol 131 | 336327 | Medicarpin | 49.22 | 0.34 | 35 | 0.017 | 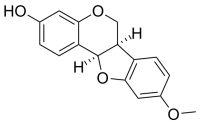 |
| Mol 134 | 72344 | Nobiletin | 61.67 | 0.52 | 35 | 0.031 | 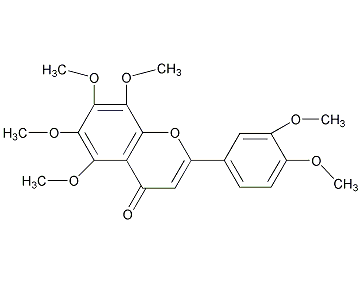 |
| Mol 157 | 5280794 | Stigmasterol | 43.83 | 0.76 | 35 | 0.036 | 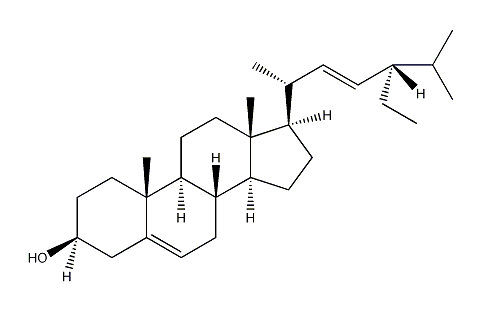 |
| Mol 113 | 5318998 | Licochalcone a | 40.79 | 0.29 | 33 | 0.016 | 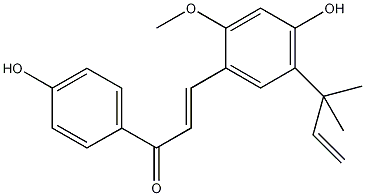 |
| Mol 17 | 9927807 | 2-[(3R)-8,8-dimethyl-3,4-dihydro-2H-pyrano[6,5-f]chromen-3-yl]-5-methoxyphenol | 36.21 | 0.52 | 32 | 0.006 | 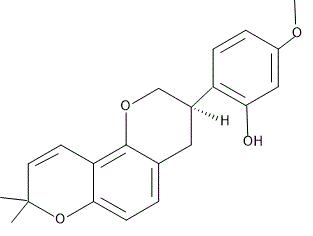 |
| Mol 158 | 177149 | Vestitol | 74.66 | 0.21 | 31 | 0.006 | 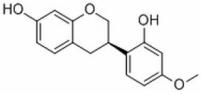 |
| Mol 151 | 10336244 | Shinpterocarpin | 80.3 | 0.73 | 31 | 0.010 | **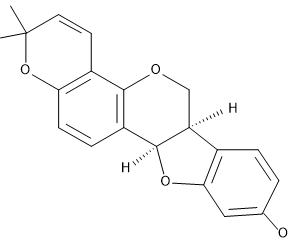** |
| Mol 111 | 15840593 | Licoagrocarpin | 58.81 | 0.58 | 30 | 0.005 | 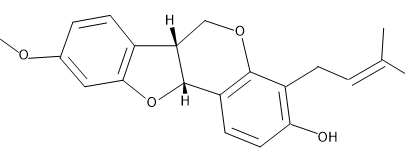 |
| Mol 13 | 480873 | 1-Methoxyphaseollidin | 69.98 | 0.64 | 30 | 0.003 | 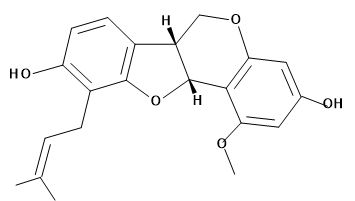 |
| Mol 22 | 15228662 | 3'-Hydroxy-4'-O-Methylglabridin | 43.71 | 0.57 | 29 | 0.003 | 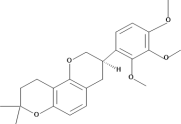 |
| Mol 23 | 5319439 | 3'-Methoxyglabridin | 46.16 | 0.57 | 29 | 0.003 | 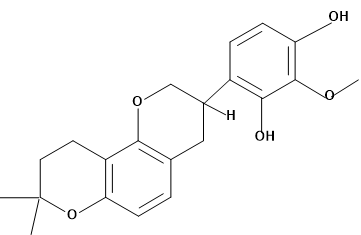 |
| Mol 97 | 3764 | HMO | 38.37 | 0.21 | 28 | 0.004 | 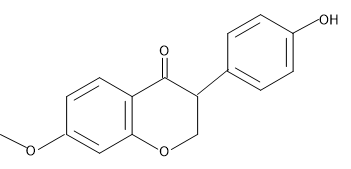 |
| Mol 84 | 5281619 | Glepidotin A | 44.72 | 0.35 | 27 | 0.002 | 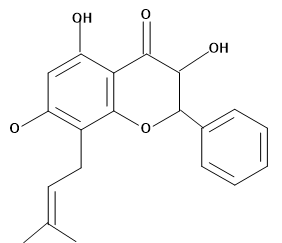 |
| Mol 93 | 5317768 | Glypallichalcone | 61.6 | 0.19 | 27 | 0.004 | 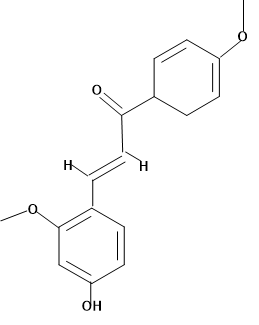 |
| Mol 89 | MOL005007 | Glyasperins M | 72.67 | 0.59 | 27 | 0.004 | 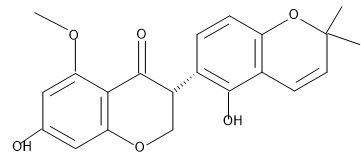 |
| Mol 36 | 5280442 | Acacetin | 34.97 | 0.24 | 27 | 0.015 | 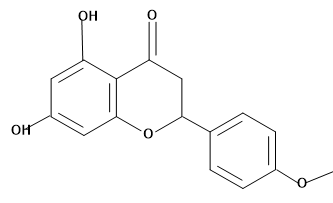 |
| Mol 32 | 268208 | 7-Acetoxy-2-methylisoflavone | 38.92 | 0.26 | 26 | 0.003 | 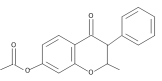 |
| Mol 132 | MOL002135 | Myricanone | 40.6 | 0.51 | 26 | 0.004 |  |
| Mol 87 | 480859 | Glyasperin C | 45.56 | 0.4 | 25 | 0.001 | 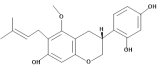 |
| Mol 19 | 10090416 | 3-(2,4-dihydroxyphenyl)-8-(1,1-dimethylprop-2-enyl)-7-hydroxy-5-methoxy-coumarin | 59.62 | 0.43 | 24 | 0.002 | 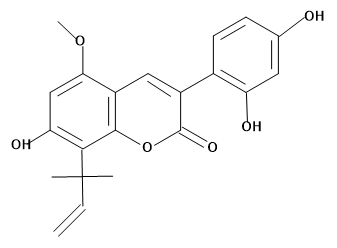 |
| Mol 142 | 162412 | Phaseolinisoflavan | 32.01 | 0.45 | 23 | 0.001 | 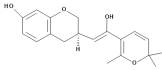 |
| Mol 46 | 5280448 | Calycosin | 47.75 | 0.24 | 23 | 0.002 | 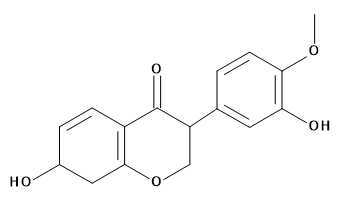 |
| Mol 75 | 5317479 | Gancaonin B | 48.79 | 0.45 | 23 | 0.001 | 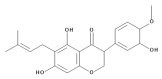 |
| Mol 95 | 73299 | hederagenin | 36.91 | 0.75 | 23 | 0.019 | 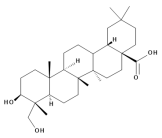 |
| Mol 110 | 15380912 | kanzonols W | 50.48 | 0.52 | 22 | 0.001 | 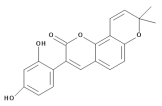 |
| Mol 126 | 5317480 | Lupiwighteone | 51.64 | 0.37 | 22 | 0.001 | 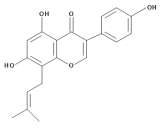 |
| Mol 83 | 5317652 | Glabrone | 52.51 | 0.5 | 22 | 0.001 | 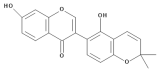 |
| Mol 7 | 637112 | (2S)-6-(2,4-dihydroxyphenyl)-2-(2-hydroxypropan-2-yl)-4-methoxy-2,3-dihydrofuro[3,2-g] chromen-7-one | 60.25 | 0.63 | 22 | 0.001 | 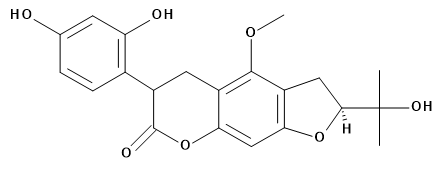 |
| Mol 43 | 5280489 | beta-carotene | 37.18 | 0.58 | 22 | 0.018 | 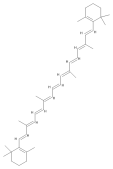 |
| Mol 27 | 14604078 | 5,7-dihydroxy-3-(4-methoxyphenyl)-8-(3-methylbut-2-enyl) chromone | 30.49 | 0.41 | 21 | 0.001 | 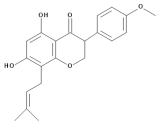 |
| Mol 9 | 10881804 | (E)-1-(2,4-dihydroxyphenyl)-3-(2,2-dimethylchromen-6-yl) prop-2-en-1-one | 39.62 | 0.35 | 21 | 0.002 | 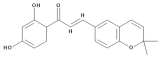 |
| Mol 135 | 13965473 | Odoratin | 49.95 | 0.3 | 21 | 0.001 | 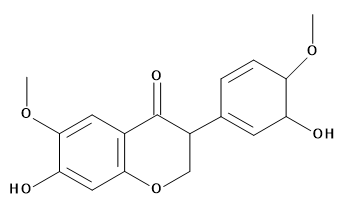 |
| Mol 74 | 5317478 | Gancaonin A | 51.08 | 0.4 | 21 | 0.001 | 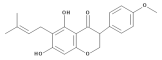 |
| Mol 117 | 392443 | licoisoflavanone | 52.47 | 0.54 | 21 | 0.001 | 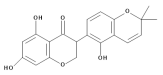 |
| Mol 76 | 480780 | Gancaonin G | 60.44 | 0.39 | 21 | 0.001 | 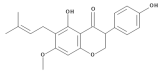 |
| Mol 61 | 5317300 | Eurycarpin A | 43.28 | 0.37 | 20 | 0.001 | 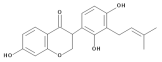 |
| Mol 81 | 480774 | Glabrene | 46.27 | 0.44 | 20 | 0.001 | 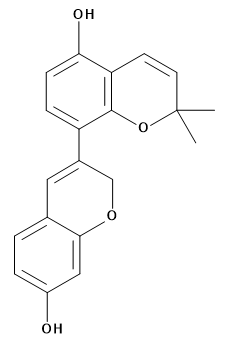 |
| Mol 114 | 5318999 | Licochalcone B | 76.76 | 0.19 | 20 | 0.002 | 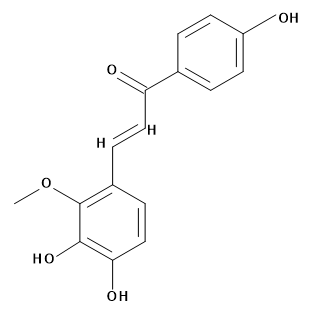 |
| Mol 138 | 19009 | palmatine | 64.6 | 0.65 | 20 | 0.002 | 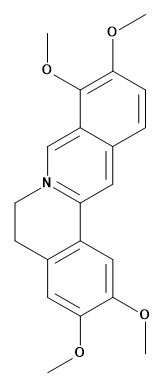 |
| Mol 57 | MOL001002 | ellagic acid | 43.06 | 0.43 | 20 | 0.016 | 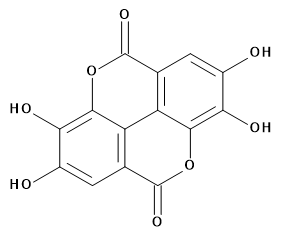 |
| Mol 54 | MOL005020 | dehydroglyasperins C | 53.82 | 0.37 | 19 | 0.001 | 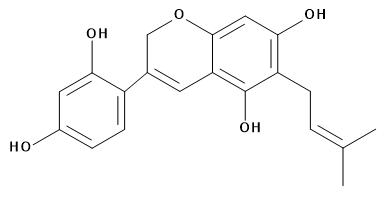 |
| Mol 112 | 636883 | Licoagroisoflavone | 57.28 | 0.49 | 19 | 0.001 | 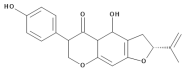 |
| Mol 94 | 5317777 | Glyzaglabrin | 61.07 | 0.35 | 19 | 0.001 | 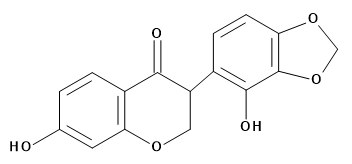 |
| Mol 20 | 14604077 | 3-(3,4-dihydroxyphenyl)-5,7-dihydroxy-8-(3-methylbut-2-enyl) chromone | 66.37 | 0.41 | 19 | 0.001 | 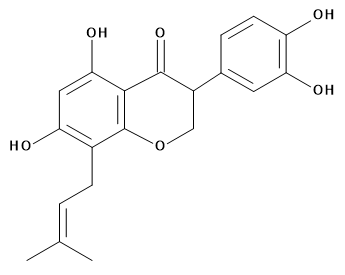 |
| Mol 88 | MOL004810 | glyasperin F | 75.84 | 0.54 | 19 | 0.001 | 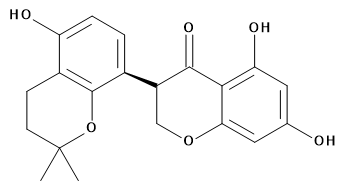 |
| Mol 119 | 5481234 | Licoisoflavone B | 38.93 | 0.55 | 18 | 0.001 | 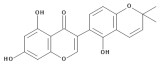 |
| Mol 92 | 5317765 | Glycyrrhiza flavonol A | 41.28 | 0.6 | 18 | 0.001 | 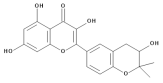 |
| Mol 149 | 5316900 | Quercetin der. | 46.45 | 0.33 | 18 | 0.001 | 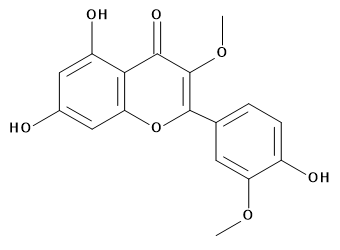 |
| Mol 150 | 5481948 | Semilicoisoflavone B | 48.78 | 0.55 | 18 | 0.001 | 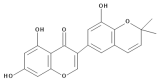 |
| Mol 115 | 49856081 | licochalcone G | 49.25 | 0.32 | 18 | 0.001 | 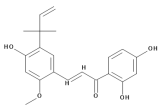 |
| Mol 90 | 480787 | Glycyrin | 52.61 | 0.47 | 18 | 0.001 | 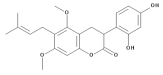 |
| Mol 100 | 91510 | Inermine | 75.18 | 0.54 | 18 | 0.004 | 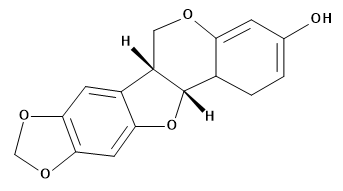 |
| Mol 39 | 158311 | Areapillin | 48.96 | 0.41 | 18 | 0.002 | 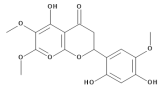 |
| Mol 42 | MOL001454 | berberine | 36.86 | 0.78 | 18 | 0.004 | 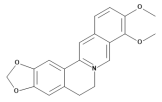 |
| Mol 14 | 14604081 | 2-(3,4-dihydroxyphenyl)-5,7-dihydroxy-6-(3-methylbut-2-enyl) chromone | 44.15 | 0.41 | 17 | 0.001 | 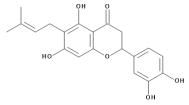 |
| Mol 120 | 122851 | licopyranocoumarin | 80.36 | 0.65 | 17 | 0.001 | 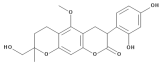 |
| Mol 101 | 5318437 | Inflacoumarin A | 39.71 | 0.33 | 16 | 0.001 | 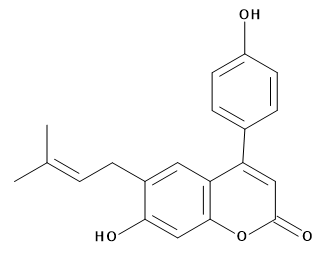 |
| Mol 104 | 5318585 | Isolicoflavonol | 45.17 | 0.42 | 16 | 0.001 | 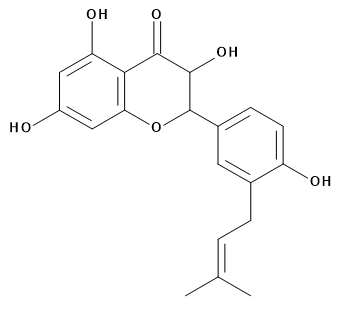 |
| Mol 121 | 5319013 | Licoricone | 63.58 | 0.47 | 16 | 0.001 | 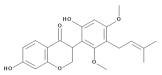 |
| Mol 4 | 928837 | (2R)-7-hydroxy-2-(4-hydroxyphenyl) chroman-4-one | 71.12 | 0.18 | 16 | 0.002 | 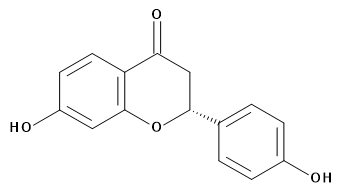 |
| Mol 30 | 25015742 | 7,2',4'-trihydroxy－5-methoxy-3－arylcoumarin | 83.71 | 0.27 | 16 | 0.001 | 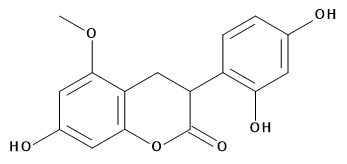 |
| Mol 106 | 5318679 | Isotrifoliol | 31.94 | 0.42 | 15 | 0.001 | 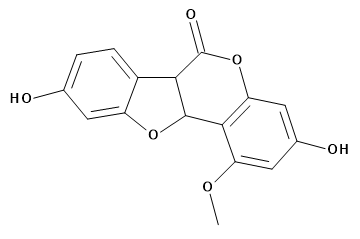 |
| Mol 141 | 44257530 | Phaseol | 78.77 | 0.58 | 15 | 0.001 | 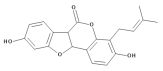 |
| Mol 107 | 5318869 | Jaranol | 50.83 | 0.29 | 14 | 0 | 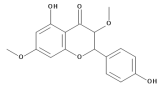 |
| Mol 73 | 6992099 | Gamma-Aminobutyric Acid | 24.09 | 0.01 | 14 | 0.03 | 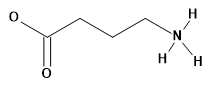 |
| Mol 6 | 197678 | (2S)-2-[4-hydroxy-3-(3-methylbut-2-enyl) phenyl]-8,8-dimethyl-2,3-dihydropyrano[2,3-f] chromen-4-one | 31.79 | 0.72 | 13 | 0 | 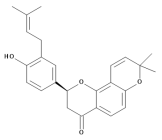 |
| Mol 8 | 193679 | (2S)-7-hydroxy-2-(4-hydroxyphenyl)-8-(3-methylbut-2-enyl) chroman-4-one | 36.57 | 0.32 | 13 | 0 | 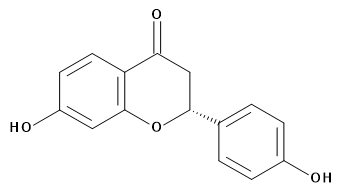 |
| Mol 10 | 11267805 | (E)-3-[3,4-dihydroxy-5-(3-methylbut-2-enyl) phenyl]-1-(2,4-dihydroxyphenyl) prop-2-en-1-one | 46.27 | 0.31 | 13 | 0 | 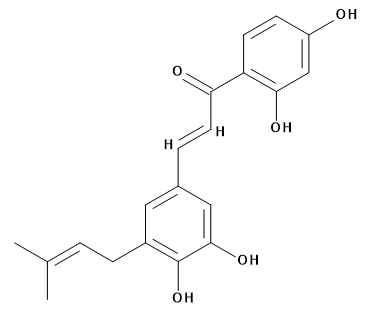 |
| Mol 77 | 5481949 | Gancaonin H | 50.1 | 0.78 | 13 | 0.001 | 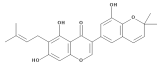 |
| Mol 21 | 389001 | 3,5,6,7-tetramethoxy-2-(3,4,5-trimethoxyphenyl) chromone | 31.97 | 0.59 | 13 | 0.001 | 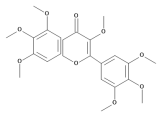 |
| Mol 85 | 442411 | Glepidotin B | 64.46 | 0.34 | 12 | 0 | 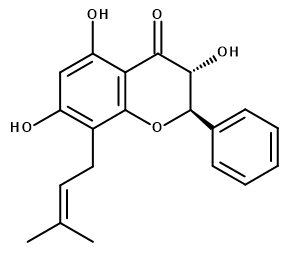 |
| Mol 91 | 5320083 | Glycyrol | 90.78 | 0.67 | 12 | 0 | 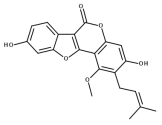 |
| Mol 58 | MOL002897 | epiberberine | 43.09 | 0.78 | 12 | 0.003 | 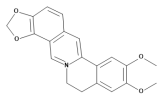 |
| Mol 3 | 9064 | (+)-catechin | 54.83 | 0.24 | 12 | 0.005 | 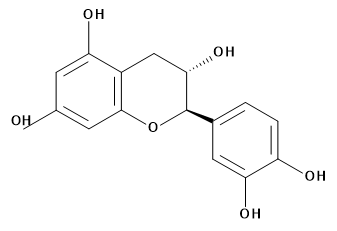 |
| Mol 60 | MOL004806 | euchrenone | 30.29 | 0.57 | 11 | 0.005 | 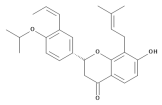 |
| Mol 12 | 11558452 | 1,3-dihydroxy-9-methoxy-6-benzofurano[3,2-c] chromenone | 48.14 | 0.43 | 11 | 0 | 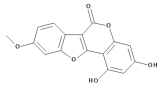 |
| Mol 80 | 124049 | Glabranin | 52.9 | 0.31 | 11 | 0 | 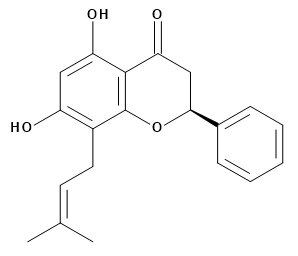 |
| Mol 82 | 124052 | Glabridin | 53.25 | 0.47 | 11 | 0 | 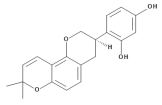 |
| Mol 86 | 480784 | glyasperin B | 65.22 | 0.44 | 11 | 0 | 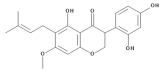 |
| Mol 11 | 11602329 | 1,3-dihydroxy-8,9-dimethoxy-6-benzofurano[3,2-c]  chromenone | 62.9 | 0.53 | 10 | 0 | 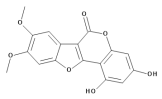 |
| Mol 96 | 72281 | Hesperetin | 70.31 | 0.27 | 10 | 0.001 | 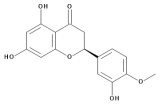 |
| Mol 102 | MOL003847 | Inophyllum E | 38.81 | 0.85 | 10 | 0.001 | 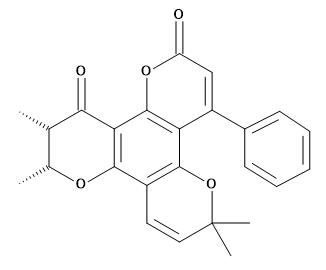 |
| Mol 52 | MOL001458 | coptisine | 30.67 | 0.86 | 10 | 0.001 | 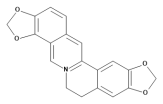 |
| Mol 109 | MOL004988 | Kanzonol F | 32.47 | 0.89 | 9 | 0 | 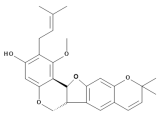 |
| Mol 29 | MOL004989 | 6-prenylated eriodictyol | 39.22 | 0.41 | 9 | 0 | 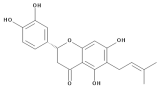 |
| Mol 35 | MOL004993 | 8-prenylated eriodictyol | 53.79 | 0.4 | 9 | 0 | 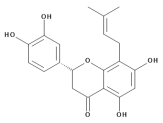 |
| Mol 161 | MOL005018 | Xambioona | 54.85 | 0.87 | 9 | 0 | 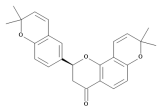 |
| Mol 140 | MOL000490 | petunidin | 30.05 | 0.31 | 9 | 0 | 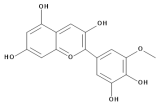 |
| Mol 122 | MOL002695 | lignan | 43.32 | 0.65 | 9 | 0.001 | 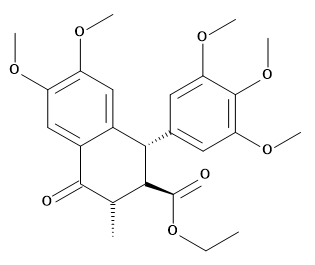 |
| Mol 24 | MOL001368 | 3-O-p-coumaroylquinic acid | 37.63 | 0.29 | 9 | 0.002 | 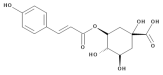 |
| Mol 64 | MOL001340 | GA120 | 84.85 | 0.45 | 9 | 0.002 |  |
| Mol 116 | 503731 | Licocoumarone | 33.21 | 0.36 | 8 | 0 |  |
| Mol 103 | MOL004948 | Isoglycyrol | 44.7 | 0.84 | 8 | 0 |  |
| Mol 38 | 656516 | Amygdalin | 4.42 | 0.61 | 8 | 0.001 |  |
| Mol 78 | MOL001358 | gibberellin 7 | 73.8 | 0.5 | 8 | 0.001 |  |
| Mol 152 | 73205 | Sigmoidin-B | 34.88 | 0.41 | 7 | 0 |  |
| Mol 118 | 5281789 | Licoisoflavone | 41.61 | 0.42 | 7 | 0 |  |
| Mol 124 | 503737 | liquiritin | 65.69 | 0.74 | 7 | 0.001 |  |
| Mol 2 | 5319252 | (+)-Anomalin | 46.06 | 0.66 | 7 | 0 |  |
| Mol 67 | MOL001352 | GA54 | 64.21 | 0.53 | 7 | 0.001 |  |
| Mol 41 | 64982 | Baicalin | 40.12 | 0.75 | 6 | 0.001 |  |
| Mol 69 | MOL001355 | GA63 | 65.54 | 0.54 | 6 | 0.001 |  |
| Mol 49 | 91520 | Catalpol | 5.07 | 0.44 | 5 | 0.002 |  |
| Mol 139 | 160179 | Perlolyrine | 65.95 | 0.27 | 5 | 0.001 |  |
| Mol 137 | MOL001924 | paeoniflorin | 53.87 | 0.79 | 5 | 0.009 |  |
| Mol 98 | MOL002690 | hydroxysafflor-yellow-A | 4.77 | 0.68 | 5 | 0.005 |  |
| Mol 62 | 6037 | FA | 68.96 | 0.71 | 4 | 0 |  |
| Mol 25 | 10237057 | 4-[(E)-4-(3,5-dimethoxy-4-oxo-1-cyclohexa-2,5-dienylidene) but-2-enylidene]-2,6-dimethoxycyclohexa-2,5-dien-1-one | 48.47 | 0.36 | 4 | 0 |  |
| Mol 1 | 23724664 | (-)-Medicocarpin | 40.99 | 0.95 | 3 | 0 |  |

**Table S5.** Docking result of 18 target proteins with 91 potential compounds

| **Proteins** | **PDB ID** | **Compounds** | **PubChem CID** | **Libdock Score** |
| --- | --- | --- | --- | --- |
| ACHE | 4EY7 | (-)-Medicocarpin | 23724664 | 175.417 |
| ACHE | 4EY7 | glyasperin B | 480784 | 147.311 |
| ACHE | 4EY7 | Glycyrrhiza flavonol A | 5317765 | 147.275 |
| ACHE | 4EY7 | Gancaonin A | 5317478 | 145.425 |
| ACHE | 4EY7 | Glyasperins M | MOL005007 | 144.871 |
| ACHE | 4EY7 | quercetin | 5280343 | 142.227 |
| ACHE | 4EY7 | (2S)-6-(2,4-dihydroxyphenyl)-2-(2-hydroxypropan-2-yl)-4-methoxy-2,3-dihydrofuro[3,2-g]chromen-7-one | 637112 | 140.367 |
| ACHE | 4EY7 | 2-[(3R)-8,8-dimethyl-3,4-dihydro-2H-pyrano[6,5-f]chromen-3-yl]-5-methoxyphenol | 9927807 | 139.462 |
| ACHE | 4EY7 | Glyasperin C | 480859 | 138.443 |
| ACHE | 4EY7 | Licoagrocarpin | 15840593 | 138.175 |
| ACHE | 4EY7 | 3'-Methoxyglabridin | 5319439 | 137.597 |
| ACHE | 4EY7 | Semilicoisoflavone B | 5481948 | 137.272 |
| ACHE | 4EY7 | Phaseolinisoflavan | 162412 | 137.139 |
| ACHE | 4EY7 | Glabridin | 124052 | 136.585 |
| ACHE | 4EY7 | kaempferol | 5280863 | 132.127 |
| ACHE | 4EY7 | Licoisoflavone B | 5481234 | 131.156 |
| ACHE | 4EY7 | isorhamnetin | 5281654 | 130.315 |
| ACHE | 4EY7 | Glabrone | 5317652 | 127.258 |
| ACHE | 4EY7 | licopyranocoumarin | 122851 | 126.154 |
| ACHE | 4EY7 | formononetin | 5280378 | 112.06 |
| ACHE | 4EY7 | 7-Acetoxy-2-methylisoflavone | 268208 | 111.421 |
| ACHE | 4EY7 | 7-Methoxy-2-methyl isoflavone | 354368 | 108.401 |
| ACHE | 4EY7 | 3,5,6,7-tetramethoxy-2-(3,4,5-trimethoxyphenyl) chromone | 389001 | 106.017 |
| AKT1 | 5KCV | baicalein | 5281605 | 118.341 |
| AKT1 | 5KCV | kaempferol | 5280863 | 118.484 |
| AKT1 | 5KCV | luteolin | 5280445 | 118.801 |
| AKT1 | 5KCV | naringenin | 439246 | 116.477 |
| AKT1 | 5KCV | quercetin | 5280343 | 123.789 |
| AKT1 | 5KCV | wogonin | 5281703 | 112.68 |
| BACE1 | 5T1U | euchrenone | MOL004806 | 108.115 |
| CASP7 | 4JR1 | luteolin | 5280445 | 104.216 |
| CASP8 | 2Y1L | quercetin | 5280343 | 107.577 |
| CASP8 | 2Y1L | beta-sitosterol | 222284 | 99.6012 |
| CASP8 | 2Y1L | acacetin | 5280442 | 97.246 |
| CDK1 | 5HQ0 | baicalein | 5281605 | 111.486 |
| CDK1 | 5HQ0 | kaempferol | 5280863 | 105.85 |
| CDK1 | 5HQ0 | quercetin | 5280343 | 112.701 |
| CHRNA7 | 5AFH | beta-sitosterol | 222284 | 124.217 |
| CHRNA7 | 5AFH | GA120 | MOL001340 | 99.0956 |
| CHRNA7 | 5AFH | GA63 | MOL001355 | 100.996 |
| CHRNA7 | 5AFH | Medicarpin | 336327 | 99.1753 |
| CHRNA7 | 5AFH | shinpterocarpin | 10336244 | 111.752 |
| CHRNA7 | 5AFH | Stigmasterol | 5280794 | 118.547 |
| CTSD | 4OBZ | Gamma-Aminobutyric Acid | 6992099 | 59.1748 |
| CTSD | 4OBZ | quercetin | 5280343 | 102.593 |
| F2 | 1AWH | Semilicoisoflavone B | 5481948 | 113.389 |
| F2 | 1AWH | quercetin | 5280343 | 111.159 |
| F2 | 1AWH | Phaseol | 44257530 | 132.073 |
| F2 | 1AWH | Perlolyrine | 160179 | 109.069 |
| F2 | 1AWH | nobiletin | 72344 | 75.8734 |
| F2 | 1AWH | Myricanone | 161748 | 91.9022 |
| F2 | 1AWH | Lupiwighteone | 5317480 | 129.768 |
| F2 | 1AWH | Licoricone | 5319013 | 127.943 |
| F2 | 1AWH | licopyranocoumarin | 122851 | 110.742 |
| F2 | 1AWH | Licoagroisoflavone | 636883 | 125.427 |
| F2 | 1AWH | Licoagrocarpin | 15840593 | 135.24 |
| F2 | 1AWH | kaempferol | 5280863 | 110.127 |
| F2 | 1AWH | isorhamnetin | 5281654 | 108.75 |
| F2 | 1AWH | Isolicoflavonol | 5318585 | 126.391 |
| F2 | 1AWH | Inflacoumarin A | 5318437 | 114.533 |
| F2 | 1AWH | Glycyrol | 5320083 | 137.766 |
| F2 | 1AWH | Glycyrin | 480787 | 137.092 |
| F2 | 1AWH | Glyasperin C | 480859 | 136.166 |
| F2 | 1AWH | glyasperin B | 480784 | 143.003 |
| F2 | 1AWH | Glepidotin A | 5281619 | 125.66 |
| F2 | 1AWH | Glabrone | 5317652 | 130.123 |
| F2 | 1AWH | Gancaonin G | 480780 | 130.925 |
| F2 | 1AWH | Gancaonin B | 5317479 | 140.718 |
| F2 | 1AWH | Gancaonin A | 5317478 | 130.334 |
| F2 | 1AWH | Eurycarpin A | 5317300 | 134.555 |
| F2 | 1AWH | Areapillin | 158311 | 112.09 |
| F2 | 1AWH | 7-Methoxy-2-methyl isoflavone | 354368 | 110.168 |
| F2 | 1AWH | 7-Acetoxy-2-methylisoflavone | 268208 | 112.243 |
| F2 | 1AWH | 4-[(E)-4-(3,5-dimethoxy-4-oxo-1-cyclohexa-2,5-dienylidene)but-2-enylidene]-2,6-dimethoxycyclohexa-2,5-dien-1-one | 10237057 | 98.7129 |
| F2 | 1AWH | 3-(3,4-dihydroxyphenyl)-5,7-dihydroxy-8-(3-methylbut-2-enyl)chromone | 14604077 | 135.246 |
| F2 | 1AWH | 3-(2,4-dihydroxyphenyl)-8-(1,1-dimethylprop-2-enyl)-7-hydroxy-5-methoxy-coumarin | 10090416 | 121.914 |
| F2 | 1AWH | 3,5,6,7-tetramethoxy-2-(3,4,5-trimethoxyphenyl)chromone | 389001 | 91.408 |
| F2 | 1AWH | 2-(3,4-dihydroxyphenyl)-5,7-dihydroxy-6-(3-methylbut-2-enyl)chromone | 14604081 | 128.173 |
| F2 | 1AWH | 1-Methoxyphaseollidin | 480873 | 125.394 |
| F2 | 1AWH | (2S)-6-(2,4-dihydroxyphenyl)-2-(2-hydroxypropan-2-yl)-4-methoxy-2,3-dihydrofuro[3,2-g]chromen-7-one | 637112 | 115.994 |
| F7 | 1W2K | (2S)-6-(2,4-dihydroxyphenyl)-2-(2-hydroxypropan-2-yl)-4-methoxy-2,3-dihydrofuro[3,2-g]chromen-7-one | 637112 | 117.479 |
| F7 | 1W2K | 2-(3,4-dihydroxyphenyl)-5,7-dihydroxy-6-(3-methylbut-2-enyl)chromone | 14604081 | 126.344 |
| F7 | 1W2K | 3'-Hydroxy-4'-O-Methylglabridin | 15228662 | 125.283 |
| F7 | 1W2K | 3'-Methoxyglabridin | 5319439 | 123.933 |
| F7 | 1W2K | 3,5,6,7-tetramethoxy-2-(3,4,5-trimethoxyphenyl)chromone | 389001 | 108.525 |
| F7 | 1W2K | 3-(2,4-dihydroxyphenyl)-8-(1,1-dimethylprop-2-enyl)-7-hydroxy-5-methoxy-coumarin | 10090416 | 118.06 |
| F7 | 1W2K | 6-prenylated eriodictyol | MOL004989 | 143.478 |
| F7 | 1W2K | 8-prenylated eriodictyol | MOL004993 | 134.437 |
| F7 | 1W2K | Areapillin | 158311 | 114.35 |
| F7 | 1W2K | Gancaonin B | 5317479 | 138.379 |
| F7 | 1W2K | Glepidotin A | 5281619 | 118.764 |
| F7 | 1W2K | glyasperin B | 480784 | 155.021 |
| F7 | 1W2K | Glyasperins M | MOL005007 | 121.466 |
| F7 | 1W2K | Glycyrrhiza flavonol A | 5317765 | 125.431 |
| F7 | 1W2K | isorhamnetin | 5281654 | 115.483 |
| F7 | 1W2K | kaempferol | 5280863 | 112.893 |
| F7 | 1W2K | licopyranocoumarin | 122851 | 128.49 |
| F7 | 1W2K | liquiritin | 503737 | 146.741 |
| F7 | 1W2K | palmatine | 19009 | 120.323 |
| F7 | 1W2K | quercetin | 5280343 | 122.234 |
| F7 | 1W2K | Semilicoisoflavone B | 5481948 | 122.99 |
| GSK3B | 5K5N | FA | 6037 | 133.879 |
| GSK3B | 5K5N | (2S)-2-[4-hydroxy-3-(3-methylbut-2-enyl)phenyl]-8,8-dimethyl-2,3-dihydropyrano[2,3-f]chromen-4-one | 197678 | 127.813 |
| GSK3B | 5K5N | 3-(3,4-dihydroxyphenyl)-5,7-dihydroxy-8-(3-methylbut-2-enyl)chromone | 14604077 | 116.573 |
| GSK3B | 5K5N | Licocoumarone | 503731 | 116.534 |
| GSK3B | 5K5N | Phaseol | 44257530 | 116.048 |
| GSK3B | 5K5N | 2-[(3R)-8,8-dimethyl-3,4-dihydro-2H-pyrano[6,5-f]chromen-3-yl]-5-methoxyphenol | 9927807 | 114.902 |
| GSK3B | 5K5N | 5,7-dihydroxy-3-(4-methoxyphenyl)-8-(3-methylbut-2-enyl)chromone | 14604078 | 114.377 |
| GSK3B | 5K5N | Lupiwighteone | 5317480 | 113.938 |
| GSK3B | 5K5N | (2S)-6-(2,4-dihydroxyphenyl)-2-(2-hydroxypropan-2-yl)-4-methoxy-2,3-dihydrofuro[3,2-g]chromen-7-one | 637112 | 113.239 |
| GSK3B | 5K5N | Semilicoisoflavone B | 5481948 | 112.872 |
| GSK3B | 5K5N | 3'-Hydroxy-4'-O-Methylglabridin | 15228662 | 112.747 |
| GSK3B | 5K5N | Licoagrocarpin | 15840593 | 111.53 |
| GSK3B | 5K5N | Glepidotin B | 442411 | 110.534 |
| GSK3B | 5K5N | (E)-3-[3,4-dihydroxy-5-(3-methylbut-2-enyl)phenyl]-1-(2,4-dihydroxyphenyl)prop-2-en-1-one | 11267805 | 110.219 |
| GSK3B | 5K5N | Isolicoflavonol | 5318585 | 110.203 |
| GSK3B | 5K5N | Licoagroisoflavone | 636883 | 109.885 |
| GSK3B | 5K5N | licochalcone a | 5318998 | 109.747 |
| GSK3B | 5K5N | Glyasperins M | MOL005007 | 109.143 |
| GSK3B | 5K5N | Gancaonin A | 5317478 | 108.865 |
| GSK3B | 5K5N | 1-Methoxyphaseollidin | 480873 | 107.66 |
| GSK3B | 5K5N | Gancaonin G | 480780 | 107.651 |
| GSK3B | 5K5N | (E)-1-(2,4-dihydroxyphenyl)-3-(2,2-dimethylchromen-6-yl)prop-2-en-1-one | 10881804 | 107.421 |
| GSK3B | 5K5N | Isoglycyrol | MOL004948 | 106.033 |
| GSK3B | 5K5N | Eurycarpin A | 5317300 | 105.157 |
| GSK3B | 5K5N | Glycyrol | 5320083 | 104.727 |
| GSK3B | 5K5N | Glycyrrhiza flavonol A | 5317765 | 104.157 |
| GSK3B | 5K5N | Odoratin | 13965473 | 103.649 |
| GSK3B | 5K5N | Inophyllum E | 5254 | 103.484 |
| GSK3B | 5K5N | glyasperin F | MOL004810 | 103.401 |
| GSK3B | 5K5N | Phaseolinisoflavan | 162412 | 102.766 |
| GSK3B | 5K5N | shinpterocarpin | 10336244 | 102.532 |
| GSK3B | 5K5N | kanzonols W | 15380912 | 102.451 |
| GSK3B | 5K5N | Glyasperin C | 480859 | 102.369 |
| GSK3B | 5K5N | Licoisoflavone B | 5481234 | 101.807 |
| GSK3B | 5K5N | Glabrone | 5317652 | 101.336 |
| GSK3B | 5K5N | Glabrene | 480774 | 100.455 |
| GSK3B | 5K5N | 3-(2,4-dihydroxyphenyl)-8-(1,1-dimethylprop-2-enyl)-7-hydroxy-5-methoxy-coumarin | 10090416 | 99.9551 |
| GSK3B | 5K5N | licochalcone G | 49856081 | 99.7084 |
| GSK3B | 5K5N | Glepidotin A | 5281619 | 99.6963 |
| GSK3B | 5K5N | petunidin | 441774 | 97.4469 |
| GSK3B | 5K5N | 1,3-dihydroxy-9-methoxy-6-benzofurano[3,2-c]chromenone | 11558452 | 97.2786 |
| GSK3B | 5K5N | 1,3-dihydroxy-8,9-dimethoxy-6-benzofurano[3,2-c]chromenone | 11602329 | 97.2585 |
| GSK3B | 5K5N | 3'-Methoxyglabridin | 5319439 | 97.2224 |
| GSK3B | 5K5N | Vestitol | 177149 | 96.9141 |
| GSK3B | 5K5N | Calycosin | 5280448 | 96.4653 |
| GSK3B | 5K5N | Isotrifoliol | 5318679 | 95.841 |
| GSK3B | 5K5N | isorhamnetin | 5281654 | 95.3654 |
| GSK3B | 5K5N | Quercetin der. | 5316900 | 92.8404 |
| GSK3B | 5K5N | HMO | 3764 | 90.6399 |
| GSK3B | 5K5N | formononetin | 5280378 | 90.4635 |
| GSK3B | 5K5N | Glyzaglabrin | 5317777 | 87.4678 |
| GSK3B | 5K5N | 7-Acetoxy-2-methylisoflavone | 268208 | 87.4515 |
| GSK3B | 5K5N | Licochalcone B | 5318999 | 87.1687 |
| GSK3B | 5K5N | 7-Methoxy-2-methyl isoflavone | 354368 | 86.1987 |
| GSK3B | 5K5N | wogonin | 5281703 | 81.7575 |
| GSK3B | 5K5N | nobiletin | 72344 | 76.2452 |
| MAPK1 | 5NGU | licochalcone a | 5318998 | 111.493 |
| MAPK1 | 5NGU | quercetin | 5280343 | 101.679 |
| MAPK1 | 5NGU | luteolin | 5280445 | 98.6004 |
| MAPK1 | 5NGU | naringenin | 439246 | 98.5039 |
| MAPK3 | 4QTB | naringenin | 439246 | 112.526 |
| NOS3 | 5UO8 | quercetin | 5280343 | 133.547 |
| NOS3 | 5UO8 | palmatine | 19009 | 115.196 |
| NOS3 | 5UO8 | Licoagrocarpin | 15840593 | 129.647 |
| NOS3 | 5UO8 | kaempferol | 5280863 | 114.707 |
| NOS3 | 5UO8 | isorhamnetin | 5281654 | 126.024 |
| NOS3 | 5UO8 | Glepidotin A | 5281619 | 134.85 |
| NOS3 | 5UO8 | Gancaonin G | 480780 | 129.22 |
| NOS3 | 5UO8 | formononetin | 5280378 | 116.52 |
| NOS3 | 5UO8 | 7-Methoxy-2-methyl isoflavone | 354368 | 106.738 |
| NOS3 | 5UO8 | 7-Acetoxy-2-methylisoflavone | 268208 | 100.122 |
| NOS3 | 5UO8 | 2-[(3R)-8,8-dimethyl-3,4-dihydro-2H-pyrano[6,5-f]chromen-3-yl]-5-methoxyphenol | 9927807 | 125.973 |
| NOS3 | 5UO8 | 1-Methoxyphaseollidin | 480873 | 141.415 |
| PRKCA | 3IW4 | beta-sitosterol | 222284 | 120.032 |
| PRKCA | 3IW4 | quercetin | 5280343 | 100.033 |
| PRKCB | 2I0E | quercetin | 5280343 | 100.719 |
| PRKCB | 2I0E | ellagic acid | MOL001002 | 91.9657 |
| PTPN1 | 5T19 | 3-(3,4-dihydroxyphenyl)-5,7-dihydroxy-8-(3-methylbut-2-enyl)chromone | 14604077 | 122.512 |
| PTPN1 | 5T19 | isorhamnetin | 5281654 | 114.335 |
| PTPN1 | 5T19 | Areapillin | 158311 | 106.186 |
| PTPN1 | 5T19 | 3-O-p-coumaroylquinic acid | MOL001368 | 99.2095 |
| PTPN1 | 5T19 | Quercetin der. | 5316900 | 98.2257 |
| PTPN1 | 5T19 | GA54 | MOL001352 | 84.3765 |
| PTPN1 | 5T19 | nobiletin | 72344 | 78.8268 |
| TNF | 5MU8 | kaempferol | 5280863 | 79.3466 |
| TNF | 5MU8 | paeoniflorin | MOL001924 | 110.373 |
| TNF | 5MU8 | quercetin | 5280343 | 104.637 |
| TNF | 5MU8 | wogonin | 5281703 | 84.6128 |

Fig. S1. The exact binding mode between active ingredients and protein targets obtained from molecule docking. (A) AKT1-quercetin, (B) CDK1-quercetin, (C) GSK3B-FA, (D) F2-glyasperin B, (E) NOS3-1-Methoxyphaseollidin, (F)ACHE-(-)-Medicocarpin.
